# Analyzing the genes and pathways related to major depressive disorder via a systems biology approach

**DOI:** 10.1002/brb3.1502

**Published:** 2019-12-25

**Authors:** Ting Fan, Ying Hu, Juncai Xin, Mengwen Zhao, Ju Wang

**Affiliations:** ^1^ School of Biomedical Engineering Tianjin Medical University Tianjin China; ^2^ Academy of Psychology and Behavior Tianjin Normal University Tianjin China

**Keywords:** major depressive disorder, network analysis, pathway cross talk

## Abstract

**Introduction:**

Major depressive disorder (MDD) is a mental disorder caused by the combination of genetic, environmental, and psychological factors. Over the years, a number of genes potentially associated with MDD have been identified. However, in many cases, the role of these genes and their relationship in the etiology and development of MDD remains unclear. Under such situation, a systems biology approach focusing on the function correlation and interaction of the candidate genes in the context of MDD will provide useful information on exploring the molecular mechanisms underlying the disease.

**Methods:**

We collected genes potentially related to MDD by screening the human genetic studies deposited in PubMed (https://www.ncbi.nlm.nih.gov/pubmed). The main biological themes within the genes were explored by function and pathway enrichment analysis. Then, the interaction of genes was analyzed in the context of protein–protein interaction network and a MDD‐specific network was built by Steiner minimal tree algorithm.

**Results:**

We collected 255 candidate genes reported to be associated with MDD from available publications. Functional analysis revealed that biological processes and biochemical pathways related to neuronal development, endocrine, cell growth and/or survivals, and immunology were enriched in these genes. The pathways could be largely grouped into three modules involved in biological procedures related to nervous system, the immune system, and the endocrine system, respectively. From the MDD‐specific network, 35 novel genes potentially associated with the disease were identified.

**Conclusion:**

By means of network‐ and pathway‐based methods, we explored the molecular mechanism underlying the pathogenesis of MDD at a systems biology level. Results from our work could provide valuable clues for understanding the molecular features of MDD.

## Graphical abstract

In this study, a systems biology approach focusing on the function correlation and interaction of the candidate genes related to major depressive disorder was adopted to explore the molecular mechanisms underlying the disease. Major pathways and the cross talks among them were detected, and novel genes potentially related to the disease were also identified.

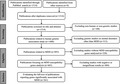

## 1 INTRODUCTION

Major depressive disorder (MDD) is a common psychiatric disorder that affects about 6% population worldwide (Kessler & Bromet, 2013; Malhi & Mann, 2018). It is estimated that the lifetime incidence of depression is 16.6% (Dunn et al., 2015), and the rate for females is twice that of males (Muglia et al., 2010). Major depressive disorder can negatively affect almost all aspects of a person, including personal life, work–life, education, and general health. At the same time, depression is a leading cause for suicide, it is estimated that 2%–8% of people diagnosed with depression die by suicide, and about 50% of people who die by suicide had depression or other mood disorders (Bachmann, 2018; Bostwick & Pankratz, 2000). The disease does not only severely limit the psychosocial functioning and deteriorate life quality of the patients, but also brings heavy spiritual and economic burden to their families and the society (Wakefield, Schmitz, Schmitz, First, & Horwitz, 2007). Actually, depression is among the most burdensome disease worldwide due to its considerable adverse effects on activities of daily living (Bruffaerts et al., 2012; Ustun, Ayuso‐Mateos, Ayuso‐Mateos, Chatterji, Mathers, & Murray, 2004). In the United States alone, depression causes about 400 million disability days per year and results in an annual economic burden as high as $210 billion (Greenberg, Fournier, Fournier, Sisitsky, Pike, & Kessler, 2015). Although in developing countries like China, the lifetime rates of depression are lower than that in developed world, the prevalence and costs related to the disease increase rapidly (Hsieh & Qin, 2018; Hu, He, He, Zhang, & Chen, 2007; Kessler & Bromet, 2013; Phillips et al., 2009; Yang et al., 2013).

Till now, the cause of MDD is still poorly understood although much effort has been dedicated to explore the pathogenesis and molecular mechanisms of the disease via various approaches (CONVERGE consortium, 2015; Flint & Kendler, 2014; Kang et al., 2012; Mehta, Menke, Menke, & Binder, 2010). Physiologically, MDD is featured with symptom heterogeneity and changes in multiple biological systems are involved (Belmaker & Agam, 2008; Guo et al., 2012). Generally, MDD develops as a result of the combination of multiple factors, including the genetic factors, environmental, and psychological factors (Han, 2012). Actually, a large fraction of the risk of MDD can be attributed to genetics (American Psychiatric Association, 2013; Kendler et al., 2019; Ripke et al., 2013). For example, it is estimated that heritability for MDD is about 40% and the risk of developing depression for members from a family with depression history is 1.5–3 times higher than the normal population (Kendler, Gatz, Gatz, Gardner, & Pedersen, 2006; Pincus et al., 1999). As a polygenic disorder with divergent genetic architecture, many genetic factors, as well as gene–environment interactions, are believed to be among the risk factors of MDD (CONVERGE consortium, 2015; Ripke et al., 2013). A number of genes have been suggested to be associated with MDD, for example, the sodium‐dependent serotonin transporter and solute carrier family 6 member 4 (SLC6A4), 5‐hydroxytryptamine receptor 2A (5HT2A), apolipoprotein E (APOE), and brain‐derived neurotrophic factor (BDNF; Bosker et al., 2011; Flint & Kendler, 2014; Lopez‐Leon et al., 2008). Among them, SLC6A4 is one of the most extensively studied genes, which is responsible for transporting serotonin from the synaptic spaces into the presynaptic neurons and recycling it in a sodium‐dependent manner. The 5‐HTTLPR polymorphism of this gene is found to be associated with both depression and other mental disorders (Clarke, Flint, Flint, Attwood, & Munafò, 2010). As the main excitatory receptor of serotonin, the genetic variants of 5HT2A have been found to be related to several psychiatric disorders, including depression (Choi et al., 2004). The epsilon‐4 type allele of APOE is found to be associated with depression in patients with Alzheimer's disease (Delano‐Wood et al., 2008). BDNF is involved in activity‐dependent neuronal plasticity, and evidence from clinical studies shows that decreased activity of BDNF occurs in the brain of patients with major depression (Lee & Kim, 2010). Similar to other complex mental disorders, genetic studies have suggested that for MDD, the individual differences may be caused by multiple genes and their variants. Genes with different functions may work cooperatively to increase the risk of MDD, with a relatively small effect exerted by each gene. In line with this view, more and more genes have been found to be potentially associated with MDD (Wray et al., 2018). For these genes, although a few plausible candidate genes have been partially replicated, some of them are considered to be problematic (Flint & Kendler, 2014). This is especially true as high‐throughput methods like genome‐wide association study (GWAS) are increasingly applied to genetic studies of the disease. Under such circumstances, a comprehensive analysis of the potential causal genes of MDD within a pathway and/or a network framework may not only provide us important insights beyond the conventional single‐gene analyses, but also offer consolidated validation for the individual candidate genes.

In the current study, we first collected the MDD‐related genes from genetic association studies. Then, we conducted biological enrichment analyses to detect the significant biological themes within these genetic factors and investigated the interactions among the enriched biochemical pathways. In addition, a MDD‐related subnetwork based on protein–protein interaction network was constructed and its topological characteristics were analyzed. This study could offer valuable hints for understanding the molecular mechanisms of MDD from a perspective of systems biology.

## 2 MATERIALS AND METHODS

### 2.1 Susceptibility gene set of MDD

**Figure 1:**
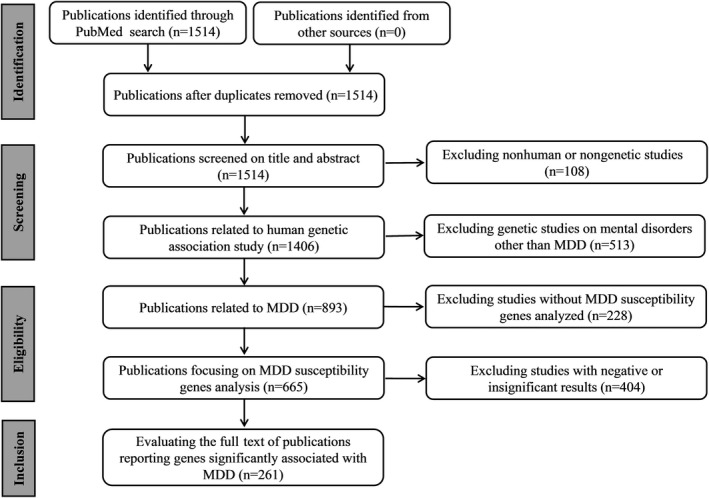
PRISMA flow diagram illustrating search strategy and studies included in the analysis. PRISMA is Preferred Reporting Items for Systematic Reviews and Meta‐Analyses (http://www.prisma-statement.org/)

As a polygenic disease, a number of genes potentially associated with the pathogenesis of MDD have been reported (Gatt, Burton, Burton, Williams, & Schofield, 2015; Manoharan, Shewade, Shewade, Rajkumar, & Adithan, 2016; Yin et al., 2016). In this study, the candidate genes for MDD were collected by searching the human genetic association studies deposited in PubMed (https://www.ncbi.nlm.nih.gov/pubmed/). Briefly, similar to previous studies (Wang & Li, 2010), we searched PubMed with the term “(Major Depressive Disorder [MeSH]) AND (Polymorphism [MeSH] OR Genotype [MeSH] OR Alleles [MeSH]) NOT (Neoplasms [MeSH]).” As of August 2017, we obtained a total of 1,514 publications related to MDD. Next, we reviewed the abstracts of these articles and kept only the association studies related to MDD with human subjects. From the selected publications, we narrowed our selection by focusing on those reporting a significant association of one or more genes with the disease. To reduce the number of potential false‐positive findings, the studies reporting negative or insignificant associations were not included although some genes analyzed in these studies might be real pathogenic genes of MDD. Then, the full reports of the selected publications were examined to ensure the consistency of the conclusions and the contents. In the collected publications, several genome‐wide association (GWA) studies on MDD were included, and genes reported to be significantly associated with MDD were selected. Via such a procedure, a list of 261 studies reporting the association of one or more candidate genes with MDD were obtained (Figure 1). From these studies, genes reported to be associated with MDD were compiled for further analysis.

### 2.2 Functional enrichment analysis

To reveal the major biological themes within the MDD‐related genes, the function characteristics of these genes were explored. Briefly, gene ontology (GO; Fu et al., 2015) and pathway enrichment analysis were conducted on the MDD‐related genes. Since in this study, we focused on the biological features underlying the candidate genes, only the GO category of biological process was analyzed. Biological pathways enriched in the MDD‐related genes may be those with disturbed function in the pathogenesis of MDD. Both GO and pathway enrichment analysis were finished by the ToppFun module of ToppGene (http://toppgene.cchmc.org; Chen, Bardes, Bardes, Aronow, & Jegga, 2009). For GO biological process analysis, items with 5 or more MDD‐related genes and a false discovery rate (FDR) less than 0.05 were kept as significantly enriched ones. Then, the enriched items were subjected to REVIGO (Supek, Bosnjak, Bosnjak, Škunca, & Šmuc, 2011; http://revigo.irb.hr/) to remove the redundant GO terms and obtained a list of nonredundant GO biological process terms enriched in the candidate genes. For pathway analysis, the Kyoto Gene and Genome Encyclopedia (Du et al., 2016; KEGG) PATHWAY was adopted as the pathway database, and a FDR threshold of 0.05 was used to define a significant pathway. False discovery rate was calculated via the method of Benjamini and Hochberg (Benjamini & Hochberg, 1995).

### 2.3 Pathway cross talk analyses

The etiology and development of a complex disease are usually the result of simultaneous disturbance of multiple biological processes or pathways. Therefore, the relationship between the pathogenically abnormal pathways can provide useful clues to understand the molecular mechanisms of the disease. Through analyzing the network formed by correlated pathways, we are able to explore the biological pathways summarized from many different studies via a systematic approach, which may help us to understand the etiology and progression of a disease from a macro perspective. Here, we used the pathways enriched in the MDD‐related genes to construct the pathway cross talk network, in which two pathways were defined as connected if they shared three or more overlapping MDD‐related genes. The purpose of such definition was to reduce the false positives and ensure that the correlation between a pathway pair was biologically meaningful. To describe the overlap between a given pair of pathways, we adopted two measurements (Jia, Kao, Kao, Kuo, & Zhao, 2011; Liu, Fan, Fan, Liu, Cheng, & Wang, 2015), that is, the Jaccard Coefficient $=\left|\frac{A\cap B}{A\cup B}\right|$ and the Overlap Coefficient$=\frac{\left|A\cap B\right|}{\min\left(\left|A\right|,\left|B\right|\right)}$, with *A* and *B* being the lists of MDD‐related genes included in the two tested pathways, and |*A*| and |*B*| representing the number of MDD‐related genes contained in the two pathways. In addition, we used the arithmetic mean of these two coefficients to measure the significance of pathway correlation and arranged all pairs of pathway in descending order of the significance. Then, Cytoscape (Shannon. et al., 2003) was used to output a diagraphic representation of the cross talk relationship between the pathways.

### 2.4 The construction of MDD subnetwork

Biomolecular network, especially the protein–protein interaction network, has become an effective tool to analyze the molecular relationship in complicated biomolecular systems (Li, Wang, Wang, Zhao, Wu, & Pan, 2016; Przulj, Wigle, Wigle, & Jurisica, 2004). In this study, we treated the genes/proteins and their interactions as nodes and edges, respectively; then, these nodes and edges were connected to form a molecular network. The protein–protein interaction network data used in this study were derived from direct physical interactions from six major common protein–protein interaction databases, that is, BioGM, Integrity, DIP, Peppermint, MIPS/Mpact, and HPRD, with the self‐interaction and redundant pairs excluded. Finally, a relatively complete human physical interaction group was obtained, which included 16,022 genes/protein and 228,122 interactions.

## 3 RESULTS

### 3.1 MDD candidate gene sets

Based on the human genetic association studies, we compiled a list of 255 candidate genes reported to be associated with MDD (Table S1; referred to as MDDgene, hereafter). Among the candidate genes collected, there were some overlapping genes that were not only associated with MDD, but also involved in the occurrence and development of other neurological diseases. For example, some genes related to immune regulation and inflammation may be associated with Alzheimer's disease or depression (e.g., IL10 and IL1B), genes of the dopamine neurotransmitter system (e.g., *DRD1* and *DRD4*), and members from the immunophilin protein family (e.g., *FKBP4* and *FKBP5*) that may be associated with Alzheimer's disease or depressive disorders. In addition, there were also genes related to the serotonin neurotransmitter system and cell transport system, such as *HTR2A*, *HTR6*, *TPH1*, *SLC1A2*, *SLC6A3*, and *SLC6A4*. At the same time, the gene set included some specific genes related to MDD, such as ADCY9, ITPR1, and PCLO, which were involved in calcium signaling, binding, and salivary secretion biological pathways. Genes related to embryonic development (e.g., CHST11 and PTPRR), cellular stress response, and blood clotting (e.g., DNAJB2, EHD3) were also included. The diversity of MDDgene was consistent with the fact that MDD was a multigene and complex disease involving various physiological procedures.

### 3.2 Functional enrichment analysis of MDDgene

Functional enrichment analysis revealed a more detailed biological function spectrum of these MDD‐related genes (Table S2). Among the GO terms overrepresented in MDDgene, those related to cell signaling, synaptic transmission, cell transport, endocrine system, or response to stimuli were included. GO terms associated with response to stimuli (e.g., multicellular organismal response to stress, response to wounding, response to light stimulus, and response to pain) were overrepresented. Such results were in line with previous findings that complicated correlations existed between the pathophysiological state of MDD and stress. Biological process terms related to synaptic transmission (e.g., trans‐synaptic signaling; synaptic signaling; neuron–neuron synaptic transmission; positive regulation of synaptic transmission; synaptic transmission, glutamatergic; and synaptic transmission, GABAergic), dopamine signaling (dopamine transport, dopamine metabolic process, dopamine uptake involved in synaptic transmission, and dopamine uptake), and other neural functions (e.g., regulation of synaptic plasticity, long‐term synaptic potentiation, neuron apoptotic process, and memory) were also enriched. Meanwhile, GO terms related to endocrine system (e.g., hormone secretion, insulin secretion, response to insulin, and response to hormone) were overrepresented. These results demonstrated that the members of MDDgene were diverse in molecular functions.

### 3.3 Pathways enriched in MDD candidate genes

Pathway analysis identified 73 pathways with significant enrichment in MDDgene (Table 1). Several pathways related to neurotransmission or neural function modulation were identified, for example, neuroactive ligand–receptor interaction, glutamatergic synapse, serotonergic synapse, dopaminergic synapse, GABAergic synapse, cholinergic synapse, and retrograde endocannabinoid signaling. A number of pathways involved in cellular signaling cascade were enriched, for example, cAMP signaling pathway, MAPK signaling pathway, and calcium signaling pathway. In addition, pathways related to neurological disorders, such as morphine addiction, amphetamine addiction, Alzheimer's disease, and alcoholism, were significantly enriched. Moreover, immune response‐associated biological processes consisting of inflammatory bowel disease, inflammatory mediator regulation of TRP channels, interleukin‐17 (IL‐17) signaling pathway, and T‐cell receptor signaling pathway were also significantly enriched, suggesting the immunological system was involved in the etiology and pathological process of MDD.

**Table 1:** Pathways enriched in MDDgene^a^

Pathways	*p* value^b^	FDR^c^	Genes included in the pathway^d^
Neuroactive ligand–receptor interaction	4.76 × 10^−18^	3.93 × 10^−15^	GABRB3, GABRD, GABRG2, AVPR1B, GHRHR, CNR1, VIPR2, DRD1, HTR1A, DRD4, HTR1B, HTR2A, HTR2C, HTR4, HTR6, GRIA1, GRIA2, GRIA4, GRIK1, GRIK4, GRIN2A, GRIN2B, NR3C1, GABBR2, GRM7, GRM8, CRHR1, CRHR2, OPRM1, P2RX7, HCRTR1, GABRA4
Glutamatergic synapse	1.53 × 10^−17^	8.43 × 10^−15^	ADCY3, ADCY6, ADCY9, ITPR1, PLD1, GNB1, GNB3, HOMER1, CACNA1A, CACNA1C, CACNA1D, GRIA1, GRIA2, GRIA4, GRIK1, GRIK4, GRIN2A, GRIN2B, GRM7, GRM8, SLC1A2, PRKCG
Serotonergic synapse	1.93 × 10^−16^	7.99 × 10^−14^	GABRB3, CYP2C19, CYP2D6, MAOA, ITPR1, GNB1, GNB3, CACNA1A, CACNA1C, CACNA1D, CACNA1S, HTR1A, HTR1B, HTR2A, HTR2C, HTR4, HTR6, SLC6A4, TPH2, PRKCG, TPH1
Morphine addiction	5.67 × 10^−16^	1.72 × 10^−13^	GABRB3, GABRD, GABRG2, PDE1C, PDE2A, PDE4B, ADCY3, ADCY6, ADCY9, GNB1, GNB3, PDE11A, CACNA1A, DRD1, GABBR2, OPRM1, ARRB1, PRKCG, GABRA4
cAMP signaling pathway	3.10 × 10^−15^	7.32 × 10^−13^	PDE4B, ADCY3, ADCY6, ADCY9, BDNF, NFKB1, AKT1, PLD1, NPY, CACNA1C, CACNA1D, VIPR2, CACNA1S, DRD1, HTR1A, HTR1B, HTR4, HTR6, GRIA1, GRIA2, GRIA4, GRIN2A, GRIN2B, GABBR2, CREB1
Dopaminergic synapse	3.76 × 10^−15^	7.77 × 10^−13^	MAOA, ITPR1, AKT1, GNB1, GNB3, CACNA1A, CACNA1C, CACNA1D, DRD1, DRD4, COMT, GRIA1, GRIA2, GRIA4, GRIN2A, GRIN2B, CREB1, GSK3B, SLC6A3, ARNTL, PRKCG
Retrograde endocannabinoid signaling	4.40 × 10^−15^	8.08 × 10^−13^	GABRB3, GABRD, GABRG2, ADCY3, ADCY6, ADCY9, ITPR1, GNB1, GNB3, CNR1, CACNA1A, CACNA1C, CACNA1D, CACNA1S, GRIA1, GRIA2, GRIA4, PRKCG, GABRA4
GABAergic synapse	1.20 × 10^−12^	1.81 × 10^−10^	GABRB3, GABRD, GABRG2, ADCY3, ADCY6, ADCY9, GNB1, GNB3, CACNA1A, CACNA1C, CACNA1D, CACNA1S, GABBR2, SLC6A1, PRKCG, GABRA4
Circadian entrainment	6.06 × 10^−11^	7.71 × 10^−9^	ADCY3, ADCY6, ADCY9, ITPR1, GNB1, GNB3, CACNA1C, CACNA1D, GRIA1, GRIA2, GRIA4, GRIN2A, GRIN2B, CREB1, PRKCG
Amphetamine addiction	8.56 × 10^−11^	1.01 × 10^−8^	MAOA, CACNA1C, CACNA1D, DRD1, GRIA1, GRIA2, GRIA4, GRIN2A, GRIN2B, CREB1, SIRT1, SLC6A3, PRKCG
MAPK signaling pathway	3.26 × 10^−10^	3.37 × 10^−8^	CACNA2D2, BDNF, PTPRR, NFKB1, NGF, AKT1, CACNA1A, CACNA1C, CACNA1D, CACNA1E, CACNA1S, CACNB2, NTRK2, TGFB1, CACNA2D4, ARRB1, EGF, PRKCG, tumor necrosis factor (TNF), MAP3K13, IL1B, TP53
Nicotine addiction	8.21 × 10^−10^	7.14 × 10^−8^	GABRB3, GABRD, GABRG2, CACNA1A, GRIA1, GRIA2, GRIA4, GRIN2A, GRIN2B, GABRA4
Calcium signaling pathway	1.63 × 10^−9^	1.28 × 10^−7^	PDE1C, AVPR1B, ADCY3, ADCY9, ITPR1, CACNA1A, CACNA1C, CACNA1D, CACNA1E, CACNA1S, DRD1, HTR2A, HTR2C, HTR4, HTR6, GRIN2A, P2RX7, PRKCG
Dilated cardiomyopathy	3.35 × 10^−8^	2.05 × 10^−6^	CACNA2D2, ADCY3, ADCY6, ADCY9, CACNA1C, CACNA1D, CACNA1S, CACNB2, TGFB1, CACNA2D4, TNF, MYBPC3
Cholinergic synapse	4.83 × 10^−8^	2.85 × 10^−6^	ADCY3, ADCY6, ADCY9, ITPR1, AKT1, GNB1, GNB3, CACNA1A, CACNA1C, CACNA1D, CACNA1S, CREB1, PRKCG
Estrogen signaling pathway	8.81 × 10^−8^	4.87 × 10^−6^	ESR1, ADCY3, SHC3, ADCY6, ADCY9, ITPR1, AKT1, FKBP4, FKBP5, GABBR2, CREB1, OPRM1
Cocaine addiction	1.07 × 10^−7^	5.51 × 10^−6^	MAOA, BDNF, NFKB1, DRD1, GRIA2, GRIN2A, GRIN2B, CREB1, SLC6A3
Aldosterone synthesis and secretion	1.19 × 10^−7^	5.61 × 10^−6^	PDE2A, ADCY3, ADCY6, ADCY9, ITPR1, HSD3B1, CACNA1C, CACNA1D, CACNA1S, CREB1, PRKCG
Insulin secretion	1.57 × 10^−6^	5.69 × 10^−5^	ADCY3, ADCY6, ADCY9, CACNA1C, CACNA1D, CACNA1S, PCLO, CREB1, PRKCG, SNAP25
Amyotrophic lateral sclerosis (ALS)	1.96 × 10^−6^	6.75 × 10^−5^	APAF1, GRIA1, GRIA2, GRIN2A, GRIN2B, SLC1A2, TNF, TP53
Longevity regulating pathway	2.40 × 10^−6^	7.93 × 10^−5^	RPS6KB1, ADCY3, ADCY6, ADCY9, NFKB1, AKT1, PRKAG2, CREB1, SIRT1, TP53
Taste transduction	1.04 × 10^−5^	2.39 × 10^−4^	PDE1C, ADCY6, GNB3, CACNA1A, CACNA1C, HTR1A, HTR1B, GABBR2, GABRA4
Oxytocin signaling pathway	1.06 × 10^−5^	2.41 × 10^−4^	CACNA2D2, ADCY3, ADCY6, ADCY9, ITPR1, PRKAG2, CACNA1C, CACNA1D, CACNA1S, CACNB2, CACNA2D4, PRKCG
Circadian rhythm	1.11 × 10^−5^	2.48 × 10^−4^	PRKAG2, NPAS2, NR1D1, CREB1, CRY1, ARNTL
Inflammatory bowel disease (IBD)	1.27 × 10^−5^	2.65 × 10^−4^	IL10, STAT1, NFKB1, TBX21, TGFB1, TNF, IL1B, IL6
Renin secretion	1.27 × 10^−5^	2.65 × 10^−4^	PDE1C, ACE, ADCY6, ITPR1, CACNA1C, CACNA1D, CACNA1S, CREB1
Gap junction	1.68 × 10^−5^	3.32 × 10^−4^	ADCY3, ADCY6, ADCY9, ITPR1, DRD1, HTR2A, HTR2C, EGF, PRKCG
Adrenergic signaling in cardiomyocytes	3.19 × 10^−5^	5.73 × 10^−4^	CACNA2D2, ADCY3, ADCY6, ADCY9, AKT1, CACNA1C, CACNA1D, CACNA1S, CACNB2, CREB1, CACNA2D4
Alzheimer's disease	3.25 × 10^−5^	5.78 × 10^−4^	NDUFV2, ITPR1, CACNA1C, CACNA1D, CACNA1S, APAF1, GRIN2A, GRIN2B, APOE, GSK3B, TNF, IL1B
Inflammatory mediator regulation of TRP channels	3.69 × 10^−5^	6.35 × 10^−4^	ADCY3, ADCY6, ADCY9, ITPR1, NGF, HTR2A, HTR2C, PRKCG, IL1B
Purine metabolism	4.08 × 10−5	6.95 × 10^−4^	PDE1C, PDE2A, PDE4B, PDE6C, ADCY3, ADCY6, ADCY9, ADK, PDE11A, XDH, NT5C2, PDE5A
Tryptophan metabolism	5.10 × 10−5	8.18 × 10^−4^	MAOA, IDO1, IDO2, EHHADH, TPH2, TPH1
Alcoholism	5.37 × 10−5	8.46 × 10^−4^	MAOA, SHC3, BDNF, GNB1, GNB3, NPY, DRD1, NTRK2, GRIN2A, GRIN2B, CREB1, SLC6A3
Longevity regulating pathway—multiple species	7.87 × 10−5	1.22 × 10^−3^	RPS6KB1, ADCY3, ADCY6, ADCY9, AKT1, PRKAG2, SIRT1
cGMP‐PKG signaling pathway	9.88 × 10−5	1.41 × 10^−3^	PDE2A, ADCY3, ADCY6, ADCY9, ITPR1, AKT1, CACNA1C, CACNA1D, CACNA1S, CREB1, PDE5A
Long‐term potentiation	1.30 × 10^−4^	1.77 × 10^−3^	ITPR1, CACNA1C, GRIA1, GRIA2, GRIN2A, GRIN2B, PRKCG
GnRH signaling pathway	1.59 × 10^−4^	2.05 × 10^−3^	ADCY3, ADCY6, ADCY9, ITPR1, PLD1, CACNA1C, CACNA1D, CACNA1S
Drug metabolism—cytochrome P450	1.71 × 10^−4^	2.18 × 10^−3^	CYP2B6, CYP2C19, CYP2D6, MAOA, UGT2A2, UGT2A1, UGT2B4
Neurotrophin signaling pathway	1.81 × 10^−4^	2.27 × 10^−3^	SHC3, BDNF, NFKB1, NGF, AKT1, NTRK2, NTRK3, GSK3B, TP53
Phospholipase D signaling pathway	1.81 × 10^−4^	2.27 × 10^−3^	AVPR1B, ADCY3, SHC3, ADCY6, ADCY9, AKT1, PLD1, GRM7, GRM8, EGF
Vascular smooth muscle contraction	2.06 × 10^−4^	2.47 × 10^−3^	AVPR1B, ADCY3, ADCY6, ADCY9, ITPR1, CACNA1C, CACNA1D, CACNA1S, PRKCG
Thyroid hormone synthesis	2.43 × 10^−4^	2.81 × 10^−3^	ADCY3, ADCY6, ADCY9, ITPR1, GPX5, CREB1, PRKCG
Chemokine signaling pathway	2.61 × 10^−4^	2.98 × 10^−3^	ADCY3, SHC3, ADCY6, ADCY9, STAT1, NFKB1, AKT1, GNB1, GNB3, GSK3B, ARRB1
Insulin resistance	4.48 × 10^−4^	4.80 × 10^−3^	RPS6KB1, NFKB1, AKT1, PRKAG2, CREB1, GSK3B, TNF, IL6
Long‐term depression	5.03 × 10^−4^	5.33 × 10^−3^	ITPR1, CACNA1A, GRIA1, GRIA2, CRHR1, PRKCG
Apelin signaling pathway	5.46 × 10^−4^	5.57 × 10^−3^	RPS6KB1, ADCY3, ADCY6, ADCY9, ITPR1, AKT1, GNB1, PRKAG2, GNB3
ErbB signaling pathway	6.12 × 10^−4^	6.17 × 10^−3^	NRG1, RPS6KB1, SHC3, AKT1, GSK3B, EGF, PRKCG
Rap1 signaling pathway	8.72 × 10^−4^	7.75 × 10^−3^	MAGI1, ADCY3, ADCY6, ADCY9, NGF, AKT1, CNR1, GRIN2A, GRIN2B, EGF, PRKCG
Type II diabetes mellitus	1.02 × 10^−3^	8.77 × 10^−3^	CACNA1A, CACNA1C, CACNA1D, CACNA1E, TNF
Adipocytokine signaling pathway	1.06 × 10^−3^	9.09 × 10^−3^	NFKB1, AKT1, PRKAG2, NPY, POMC, TNF
Prolactin signaling pathway	1.15 × 10^−3^	9.71 × 10^−3^	ESR1, SHC3, STAT1, NFKB1, AKT1, GSK3B
AGE‐RAGE signaling pathway in diabetic complications	1.41 × 10^−3^	1.12 × 10^−2^	STAT1, NFKB1, AKT1, TGFB1, TNF, IL1B, IL6
Melanogenesis	1.58 × 10^−3^	1.23 × 10^−2^	ADCY3, ADCY6, ADCY9, POMC, CREB1, GSK3B, PRKCG
Osteoclast differentiation	1.61 × 10^−3^	1.24 × 10^−2^	SPI1, STAT1, NFKB1, AKT1, CREB1, TGFB1, TNF, IL1B
Gastric acid secretion	1.64 × 10^−3^	1.25 × 10^−2^	ADCY3, ADCY6, ADCY9, ITPR1, KCNK2, PRKCG
Ras signaling pathway	1.64 × 10^−3^	1.25 × 10^−2^	SHC3, NFKB1, NGF, AKT1, PLD1, GNB1, GNB3, GRIN2A, GRIN2B, EGF, PRKCG
FoxO signaling pathway	1.77 × 10^−3^	1.31 × 10^−2^	IL10, AKT1, PRKAG2, HOMER1, SIRT1, TGFB1, EGF, IL6
Toll‐like receptor signaling pathway	1.88 × 10^−3^	1.35 × 10^−2^	STAT1, NFKB1, AKT1, IKBKE, TNF, IL1B, IL6
Cardiac muscle contraction	2.01 × 10^−3^	1.39 × 10^−2^	CACNA2D2, CACNA1C, CACNA1D, CACNA1S, CACNB2, CACNA2D4
Regulation of lipolysis in adipocytes	2.10 × 10^−3^	1.45 × 10^−2^	ADCY3, ADCY6, ADCY9, AKT1, NPY
NOD‐like receptor signaling pathway	2.37 × 10^−3^	1.59 × 10^−2^	STAT1, ITPR1, NFKB1, NAMPT, P2RX7, IKBKE, TNF, IL1B, IL6
Steroid hormone biosynthesis	2.89 × 10^−3^	1.81 × 10^−2^	UGT2A2, UGT2A1, UGT2B4, HSD3B1, COMT
Thyroid hormone signaling pathway	3.48 × 10^−3^	2.10 × 10^−2^	ESR1, STAT1, DIO1, AKT1, GSK3B, PRKCG, TP53
Rheumatoid arthritis	4.12 × 10^−3^	2.34 × 10^−2^	ATP6V1B2, TGFB1, TNF, CTLA4, IL1B, IL6
Cytosolic DNA‐sensing pathway	4.43 × 10^−3^	2.47 × 10^−2^	NFKB1, IL33, IKBKE, IL1B, IL6
IL‐17 signaling pathway	4.84 × 10^−3^	2.67 × 10^−2^	NFKB1, GSK3B, IKBKE, TNF, IL1B, IL6
Bile secretion	6.88 × 10^−3^	3.45 × 10^−2^	ADCY3, ADCY6, ADCY9, ABCB1, UGT2B4
Hypoxia‐Inducible Factor (HIF‐1) signaling pathway	7.21 × 10^−3^	3.60 × 10^−2^	RPS6KB1, NFKB1, AKT1, EGF, PRKCG, IL6
T‐cell receptor signaling pathway	7.91 × 10^−3^	3.80 × 10^−2^	IL10, NFKB1, AKT1, GSK3B, TNF, CTLA4
Metabolism of xenobiotics by cytochrome P450	8.18 × 10^−3^	3.90 × 10^−2^	CYP2B6, CYP2D6, UGT2A2, UGT2A1, UGT2B4
Apoptosis	8.87 × 10^−3^	4.13 × 10^−2^	ITPR1, NFKB1, NGF, AKT1, APAF1, TNF, TP53
Th17 cell differentiation	9.46 × 10^−3^	4.22 × 10^−2^	STAT1, NFKB1, TBX21, TGFB1, IL1B, IL6
TNF signaling pathway	9.88 × 10^−3^	4.37 × 10^−2^	NFKB1, AKT1, CREB1, TNF, IL1B, IL6

Abbreviations: FDR, false discovery rate; IL‐17, interleukin‐17; MDD, major depressive disorder.

a MDDgene: Genes related to major depressive disorder.

b *p* value was calculated by Fisher's exact test.

c FDR was calculated by Benjamini & Hochberg (BH) method.

d Genes in MDDgene that were included in the specific pathway.

**Figure 2:**
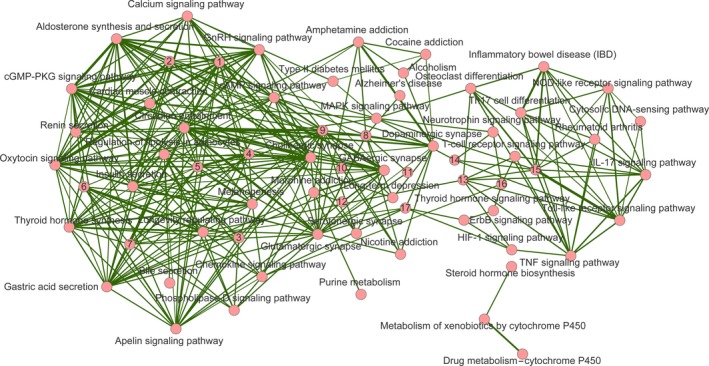
Cross talk between pathways related to major depressive disorder (MDD). The circular nodes represent pathways significantly enriched in the genes in associated with MDD, and each edge represents the cross talk between the two connected pathways, with the width corresponding to strength of the cross talk (i.e., the average of the Jaccard Coefficient and the Overlap Coefficient). The nodes labeled with numbers represent the following pathways: 1, “vascular smooth muscle contraction”; 2, “dilated cardiomyopathy”; 3, “estrogen signaling pathway”; 4, “gap junction”; 5, “inflammatory mediator regulation of TRP channels”; 6, “long‐term potentiation”; 7, “longevity regulated pathway‐multiple species”; 8, “Rap1 signaling pathway”; 9, “neuroactive ligand–receptor interaction”; 10, “amyotrophic lateral sclerosis”; 11, “taste transduction”; 12, “insulin resistance”; 13, “apoptosis”; 14, “AGE‐RAGE signaling pathway in diabetic complications”; and 15, “prolactin signaling pathway”

We further analyzed the cross talk between the enriched pathways that were significantly associated with MDD. Most of these pathways interacted with one or more other pathways, which resulted in a cross talk network with 68 nodes (i.e., pathways) and 325 edges (i.e., connection between two neighboring pathways; Figure 2). Based on the biological function and the relevance of these pathways, we could roughly divide the pathways into three modules. Pathways in the first module were mainly related to cellular signaling transduction (e.g., cAMP signaling pathway, calcium signaling pathway, cGMP‐PKG signaling pathway, and phospholipase D signaling pathway) or the endocrine control (e.g., renin secretion, aldosterone synthesis and secretion, oxytocin signaling pathway, thyroid hormone synthesis, and estrogen signaling pathway). In the second module, many pathways were related to neuronal function like neurotransmission (e.g., cholinergic synapse, dopaminergic synapse, GABAergic synapse, glutamatergic synapse, and long‐term depression), neurological disorders (e.g., amphetamine addiction, cocaine addiction, morphine addiction, nicotine addiction, alcoholism, amyotrophic lateral sclerosis, and Alzheimer's disease), endocrine, and metabolic diseases (e.g., type II diabetes mellitus and insulin resistance). The last module was largely concentrated in pathways related to the immune system, such as cytosolic DNA‐sensing pathway, IL‐17 signaling pathway, NOD‐like receptor signaling pathway, T‐cell receptor signaling pathway and Th17 cell differentiation, and Toll‐like receptor signaling pathway. These three modules were not independent of each other; instead, they were interconnected by one or more pathways. In this cross talk network, a few other types of pathways related to biological processes such as aging, apoptosis, and environmental adaptation were also included. Thus, the etiology and development of MDD could be the consequence of the abnormality in multiple systems.

### 3.4 MDD‐specific network

**Figure 3:**
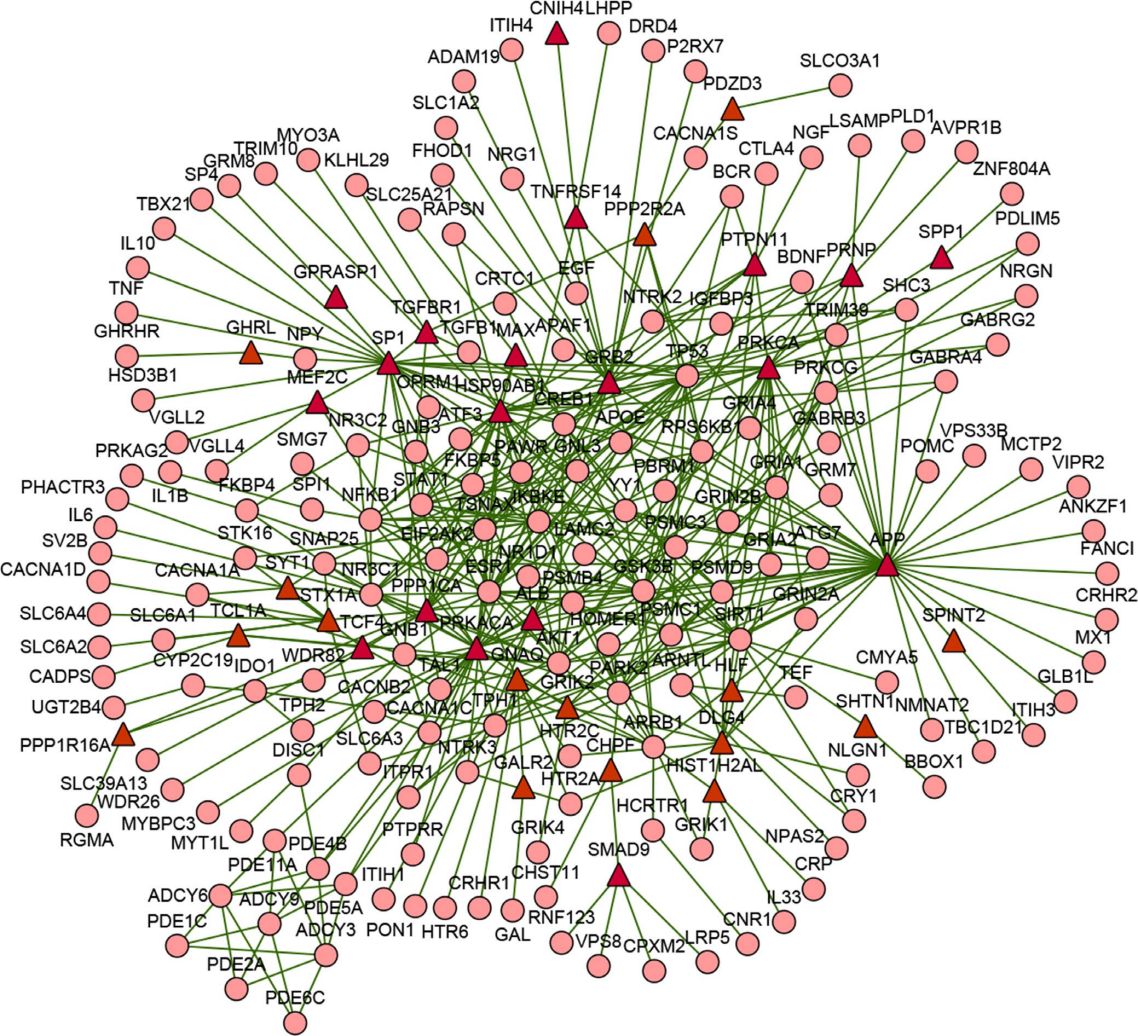
Major depressive disorder specific network. The major depressive disorder (MDD)‐specific subnetwork was constructed via the Steiner minimum algorithm, including 203 nodes and 415 edges. The circular nodes represent the known genes related to MDD, while the red triangular nodes represent the genes newly introduced to the subnetwork, which may be genes potentially related to MDD. The edge represents the interaction between genes

To further explore the feature of genes associated with MDD, we constructed a subnetwork for the disease from the human protein–protein interaction network via the Steiner minimal tree algorithm (Li, Mao, Mao, & Wei, 2008; Sadeghi & Fröhlich, 2013), which tried to connect the largest number of input nodes (genes included in MDDgene in our case) via the least number of interlinking nodes (Figure 3). The subnetwork contained 203 nodes and 415 edges (interactions between genes). Of the genes in MDDgene, 168 out of 255 were included in the MDD‐specific network, which accounted for 65.9% of MDDgene and 82.8% of the genes in the network, demonstrating a relatively high coverage of MDDgene in the subnetwork.

At the same time, 35 genes outside of MDDgene were introduced into the MDD‐specific molecular network (Table 2). Given these genes interacted closely with those known to be related to MDD, they might also be involved in the pathogenesis of the disease phenotype. Further functional enrichment analysis indicated that these genes were mainly involved in neuronal development, behavior, learning and memory, and glutamate receptor signaling.

**Table 2:** Genes included in MDD subnetwork but not in MDD gene set^a^

Gene ID	Gene symbol	Gene name
8811	GALR2	Galanin receptor 2
10653	SPINT2	Serine peptidase inhibitor, Kunitz type 2
29097	CNIH4	Cornichon family AMPA receptor auxiliary protein 4
8332	HIST1H2AL	Histone cluster 1 H2A family member l
3131	HLF	HLF, PAR bZIP transcription factor
2898	GRIK2	Glutamate ionotropic receptor kainate type subunit 2
79586	CHPF	Chondroitin polymerizing factor
51738	GHRL	Ghrelin and obestatin prepropeptide
6857	SYT1	Synaptotagmin 1
2776	GNAQ	G protein subunit alpha q
6804	STX1A	Syntaxin 1A
84988	PPP1R16A	Protein phosphatase 1 regulatory subunit 16A
8115	TCL1A	T‐cell leukemia
79849	PDZD3	PDZ domain containing 3
57698	SHTN1	Shootin 1
1742	DLG4	Disks large MAGUK scaffold protein 4
5520	PPP2R2A	Protein phosphatase 2 regulatory subunit Balpha
4208	MEF2C	Myocyte enhancer factor 2C
5578	PRKCA	Protein kinase C alpha
8764	TNFRSF14	TNF receptor superfamily member 14
9737	GPRASP1	G protein‐coupled receptor‐associated sorting protein 1
5781	PTPN11	Protein tyrosine phosphatase, nonreceptor type 11
5566	PRKACA	Protein kinase cAMP‐activated catalytic subunit alpha
5621	PRNP	Prion protein
213	ALB	Albumin
6667	SP1	Sp1 transcription factor
5499	PPP1CA	Protein phosphatase 1 catalytic subunit alpha
4093	SMAD9	SMAD family member 9
3326	HSP90AB1	Heat‐shock protein 90 alpha family class B member 1
6925	TCF4	Transcription factor 4
4149	MAX	MYC‐associated factor X
7046	TGFBR1	Transforming growth factor beta receptor 1
2885	GRB2	Growth factor receptor‐bound protein 2
6696	SPP1	Secreted phosphoprotein 1
351	APP	Amyloid beta precursor protein

Abbreviations: MDD, major depressive disorder; IL‐17, interleukin‐17.

a The collected MDD candidate genes were used as seed nodes to construct and extract potential specific disease subnetworks by introducing a minimum number of genes according to the Steiner minimum tree algorithm. Among them, 35 genes are newly introduced non‐MDD genes.

## 4 DISCUSSION

Recent years, our understanding on the molecular mechanisms of MDD has been greatly improved. With the advancement and maturity of high‐throughput technology, we are able to identify the elements related to this disease on much larger scales. Although more and more genes/proteins potentially involved in the disease have been reported, a thorough analysis of the biochemical processes associated with the pathogenesis of MDD from the molecular aspect is still missing. In such case, a systematic analysis of MDD‐related genes via a pathway‐ and network‐based analytical framework will provide us insight on the disease beyond the single candidate gene‐based analyses. In this study, we tried to pool and curate the genes related to MDD from human genetic studies, and systematically delineated the interconnection of these genes based on pathway and network analysis.

Compared with candidate gene(s)‐based approach, a comprehensive analysis on MDD‐related genes conducted in this study has its own advantages. By implementing an extensive screening and compilation of human genes from genetic association studies on MDD, we obtained valuable gene source data for further analysis. Especially, since the genetic susceptibility of MDD is related to multiple genes functioning cooperatively (Williams‐Skipp et al., 2009), it is essential to explore the biological features of genes related to MDD from a perspective of molecular network level. At the same time, by focusing on the biological correlation of genes, pathway and network analysis can not only give us a more comprehensive view for the pathological mechanisms of MDD, but they also are more robust to the influence of false‐positive genes.

As revealed by function enrichment analysis, genes related to MDD were diverse in function, mainly involved in cell signaling, immune system, metabolic process, drug response processes, and neurodevelopment. Gene ontology biological process terms such as reverse cholesterol transport, positive regulation of IL‐6 production, response to ethanol, lipoprotein metabolic process, diol metabolic process, xenobiotic metabolic process, and regulation of neuronal synaptic plasticity were overrepresented among MDDgene, implying the important roles of these processes in the pathological processes of MDD. In addition, we noticed terms related to memory, visual learning, social behavior, sleep, axon regeneration, and axon guidance were also enriched in MDDgene, consistent with a priori biological findings on MDD.

**Figure 4:**
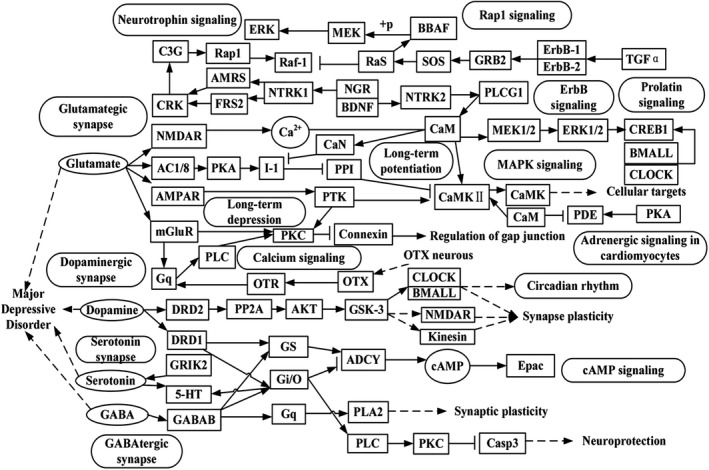
Diagram of the major pathways and genes related to major depressive disorder (MDD). MDD is a complex disease with a number of genes and pathways coordinated and interrelated by multiple systems. The nodes in the rectangle represent the genes involved in each pathway. Small elliptical nodes represent neurotransmitters such as GABA, serotonin, dopamine, and glutamate. The large ellipse represents the main pathway involved in MDD. The dashed line and the solid line represent the indirect and direct relationship between the parts; the line of the arrow or breakpoint indicates the activation and inhibition of the action, respectively

Biological pathways enriched in MDDgene were involved in multiple biological systems, including the nervous system, immune system, endocrine systems, and signal transduction systems, or related to disorders like drug addiction and immune metabolism diseases. Actually, abnormality or dysregulation of many of these pathways has been known to be related to neurological diseases. For example, calcium signaling pathway has been reported to be involved in diseases such as nicotine addiction (Wang & Li, 2010), Alzheimer's disease (Karttunen et al., 2011), bipolar disorder and schizophrenia (Berridge, 2014), and depression (Donev & Alawam, 2015; Duman & Voleti, 2012). Another example is the pathway of GABAergic synapse. As the most abundant inhibitory neurotransmitter in the mammalian central nervous system (Lloyd, Perrault, & Zivkovic, 2017; Zhang et al., 2018), the defect of GABAergic neurons in the frontal cortex may be responsible for the pathogenesis and development of MDD (Czéh et al., 2018). The identification of GABAergic synapse pathway in MDDgene provides additional evidence that GABAergic dysfunction may lead to mood and cognitive symptoms of MDD. Interleukin‐17 in the IL‐17 signaling pathway plays a crucial role in acute and chronic inflammatory responses (Zhao, Li, Li, Wang, Manthari, & Wang, 2018). Neuroendocrine and immune system interactions play an important role in stress response (Ashley & Demas, 2017; Dantzer, 2018). Such results suggest that the immune system plays important roles in the onset of MDD. Both stress and inflammatory cytokine activation have been reported to have adverse effect on the neurogenesis and neural plasticity (Syed et al., 2018). By comparing our results with that of a meta‐analysis on genes implicated in MDD (Gatt et al., 2015; Manoharan et al., 2016; Yin et al., 2016), we found that most of the pathways reported earlier were also identified in the current study. Further, as indicated by the pathway cross talk analysis, multiple physiological pathways and their interaction may be critical in the pathogenesis of MDD. Then, by integrating the result from this study and prior biological knowledge on the molecular mechanisms of MDD, we summarized a molecular network of the major pathway interaction (Figure 4). In this molecular network, some key genes and pathways work together, such as glutamate synapses, dopamine synapses, serotonin synapses, gamma‐aminobutyric acid (GABA) synapses, cAMP‐mediated signal transduction cascades and circadian rhythm, and other signaling pathways. Among them, CaM and CaMKII play an important role in long‐term potentiation and long‐term depression, and they connect multiple pathway genes, suggesting that CaM and CaMKII may play an important role in the development of synaptic plasticity. Perhaps it is the key factor that affects the development of MDD. In addition, the genes CLOCK and BMALL are essential in several pathways related to MDD (e.g., prolactin signaling and circadian rhythm), suggesting they may be involved in the development of MDD. Since these pathways are interconnected and they function cooperatively, dysfunction in one pathway may cause abnormality or dysregulation in others and eventually lead to the onset and development of MDD.

In the pathway cross talk network, there were several pathways related to other diseases, such as pathway of Alzheimer's disease, AGE‐RAGE signaling pathway in diabetic complication, pathway of alcoholism, and pathway of dilated cardiomyopathy. Available evidence shows that each of these diseases has close correlation MDD. For example, it has been found that depression is associated with an increased risk of Alzheimer's disease, with MDD patients being 1.5 times more likely to develop Alzheimer's disease and 20% to 50% patients with Alzheimer's disease having depressive symptoms (Gibson et al., 2017; Saczynski. et al., 2010). Comparison of the molecules involved in the two diseases shows that they share a number of genes, regulatory elements like miRNAs, and quite several biological processes and pathways (Hu, Xin, Xin, Hu, Zhang, & Wang, 2017; Mendes‐Silva et al., 2016), which is consistent with the prior knowledge that depression may be a risk factor for Alzheimer's disease or part of the symptoms of dementia. We also detected pathways related to diabetes (i.e., insulin secretion, insulin resistance, and type II diabetes mellitus). Connection between diabetes and depression has been studied extensively, and there is clear symbiotic relationship between the two diseases (Han, 2012; Lloyd, Pambianco, Pambianco, & Orchard, 2010; Patterson, Khazall, Khazall, MacKay, Anisman, & Abizaid, 2013; Roy & Lloyd, 2012; Semenkovich, Brown, Brown, Svrakic, & Lustman, 2015). A possible explanation is that diabetes may affect the function of brain regions like hippocampus (Semenkovich et al., 2015), the abnormality in which may be involved in the pathogenesis of MDD (Colla et al., 2007; Ho, Sommers, Sommers, & Lucki, 2013).

**Figure 5:**
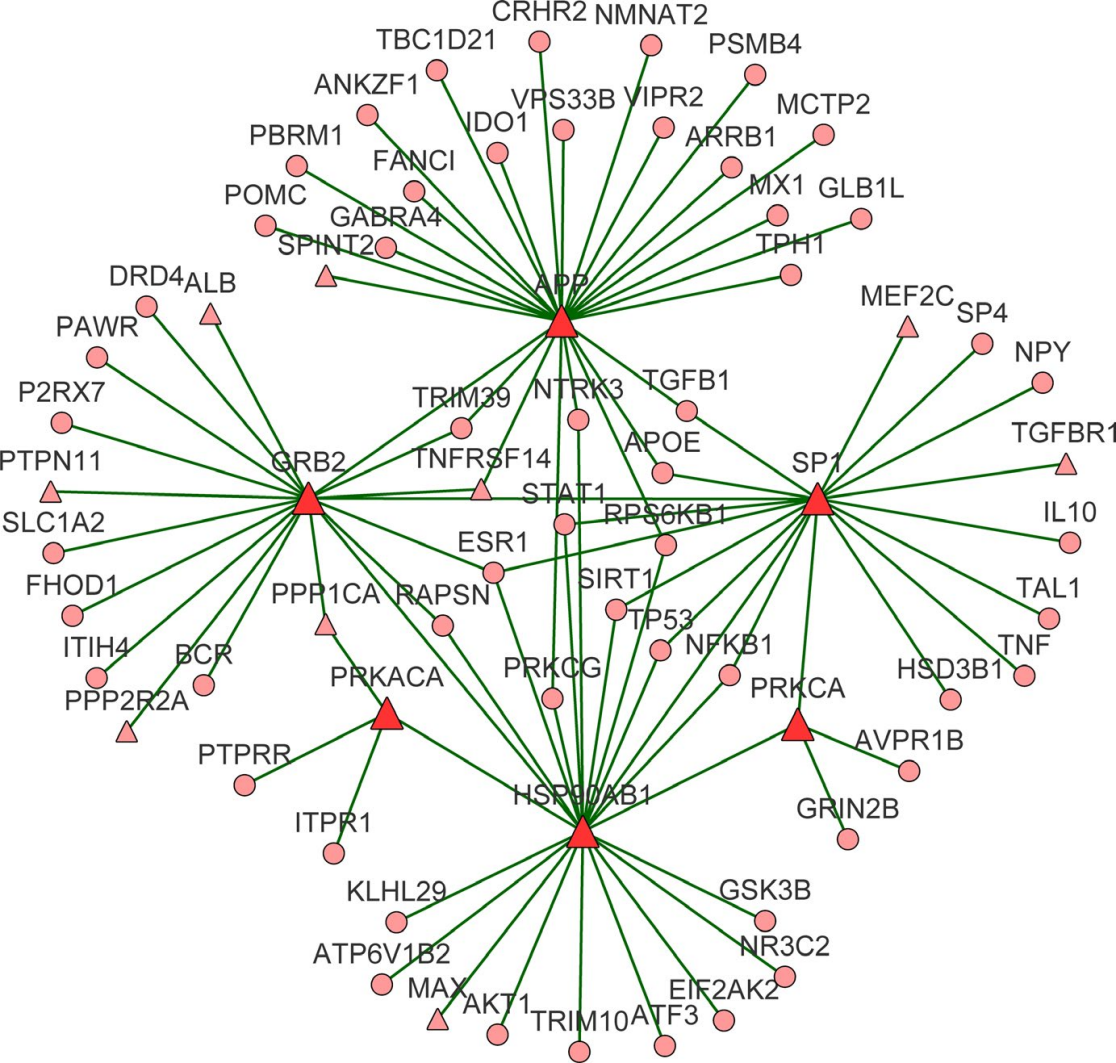
Novel genes potentially related to major depressive disorder (MDD). The six essential genes were mapped to protein‐protein interaction network (PPIN) to extract the genes interacting with them. The triangle nodes are non‐MDD susceptibility gene, and the red triangle nodes are essential genes. The small circular nodes are susceptibility genes in the MDD gene set

In the subnetwork constructed by genes related to MDD, six genes outside of the MDDgene, that is, APP (amyloid beta precursor protein), HSP90AB1 (heat‐shock protein HSP 90‐beta), PRKACA (catalytic subunit α of protein kinase A), GRB2 (growth factor receptor‐bound protein 2), PRKCA (protein kinase C alpha), and SP1 (transcription factor Sp1), were localized at the key positions in the subnetwork. Compared with other genes, they interacted with more genes in the network. We further extracted the genes interacting with these six genes to examine their connection with other genes (Figure 5). In the genetic interaction network centered on these genes, approximately 78% (54/69) of the genes were members of MDDgene. Functionally, pathways related to the immune system or the nervous system were enriched in these genes, implicating these genes may be involved in MDD through their connection with pathways related to the immune system or the nervous system.

Among these genes, APP encodes the precursor molecule of beta amyloid, the primary component of amyloid plaques found in the brains of patients with Alzheimer's disease. In our pathway enrichment analysis, pathway related to Alzheimer's disease was also enriched in MDDgene. Thus, even though the evidence on the role of APP in the pathogenesis of MDD is still limited, it may be closely related to MDD. Catalytic subunit α of protein kinase A and GRB2 have been reported to be related to MDD. Previous studies using human peripheral and postmortem brain tissue samples have shown that some depressed patient's exhibit reduced PRKACA activity (Kastenhuber. et al., 2017; Pandey et al., 2007). Growth factor receptor‐binding protein 2 (Melmed, Polonsky, Larsen, & Kronenberg, 2016) is a 217 amino acid protein containing an SH2 domain and a pair of SH3 domains that are constitutions associated with a polyphonic sequence in the SOS protein. Glombik et al. (2017) examined the effects of the antidepressant imipramine, fluoxetine, and tianeptine on the insulin signaling pathway in the brain of adult antenatal stressed rats and found that the behavioral effectiveness of antidepressant therapy may be related to the beneficial effects of antidepressants on the insulin receptor phosphorylation pathway. This result was obtained by measuring mRNA and protein expression of insulin, insulin receptor, insulin receptor substrate (IRS‐1, IRS‐2), and adaptor protein (SHC1, GRB2) before and after administration in the frontal cortex and hippocampus. In the hippocampus, it was found to have a certain relationship with the adaptor protein SHC1/GRB2. In addition, Sun et al. (2011) found that six of the seven SNPs in the GRB2 gene in the Irish population showed significant association with schizophrenia, and two of them (rs7207618 and rs9912608) remained significant after permutation test or Bonferroni correction test, indicating that GRB2 may be a risk gene for Schizophrenia in the Irish population.

Although our analyses suggest that these newly introduced genes may be involved in the pathogenesis or development of MDD, further investigation based on experiments is essential to decipher their connection with this disease.

Recently years, several models on the mechanisms of MDD have been developed. For example, based on the known regulatory network of MDD physiological pathways, Stapelberg et al. (2018) and Stapelberg, Neumann, Neumann, Shum, and Headrick (2019) proposed the psycho‐immune‐neuroendocrine network for MDD. The model mainly emphasizes the key transition forms from health to disease (MDD) state and can diagnose and predict the incidence of disease. Unlike their disease process model, our study constructed a framework for the analysis of complex disease susceptible genes based on the approach of biological pathways and protein interaction networks; more attention has been paid to the role of disease susceptible genes and their interactions in the pathogenesis of disease.

There are also some databases related to the genetic information of MDD, but no dataset specific for MDD. As MDD is a complex disease with high heterogeneity, its occurrence and development are inseparable from the interaction of components at different levels of each system. MK4MDD (Guo et al., 2012) is a database for MDD that contains data from seven different levels of research published in MDD experiments, as well as some MDD‐related genes and pathways collected through the literature. As with the genes in the database, we started by studying the literature and employed genes that have sufficient evidence to show that they are indeed related to MDD. By examining the various information in the database, we found that many items of the GO biological process and pathways enriched in the MDDgene detected in this study were also included in the database, such as behavior, learning or memory, neuron development and long‐term depression, vascular smooth muscle contraction, and type II diabetes mellitus. Thus, the gene set MDDgene built in this study is relatively reliable, which could be a useful resource for MDD study.

There are some limitations in the current study. First, there are some subjective factors in the procedure of MDD candidate gene collection. For example, the collection of studies on MDD may be not comprehensive enough because of the specific screening conditions we used; in many candidate gene studies, the selection of genes could be biased as they are often chosen based on prior knowledge of the disease itself or related diseases. For such reason, the collected MDD‐related genes may include a high fraction of genes also associated with other mental disorder, but we believe that with further improvement, the pathogenic genes for MDD will be supplemented and the dataset will become more and more reliable. Second, although multiple pathway databases are available, we only utilized the KEGG pathway database in pathway enrichment analysis, which might lead to bias in the result. But on the other hand, the definition of pathway may be different in various pathway databases, which means pathways with same or similar names may be not consistent in different databases. To avoid the potential confusion caused by merging multiple databases, we relied on KEGG pathway database for our analysis. Third, the current available human PPIN is still incomplete and may include false‐positive data, which may have impact on our results. Although there are some shortcomings in the current study, we believe the results obtained by us should be reliable. Finally, several studies on MDD via GWA meta‐analysis have been published recently (Howard et al., 2018, 2019; Wray et al., 2018). Based on large sample sizes, a number of novel variants potentially associated with MDD have been identified in each study. These studies clearly demonstrated the power of GWA meta‐analysis in detecting the genetic factors underlying complex disorders like MDD. However, due to the difficulties in data integration, we did not include the genes reported in these studies in our analysis.

## 5 CONCLUSION

In this study, we conducted a systematic analysis on genes genetically associated with MDD. Based on the 255 disease‐related genes collected, 73 significantly enriched pathways were identified. Pathway cross talk analysis indicated that three major modules were formed by these biological pathways, with each module including pathways related to cellular signaling transduction or the endocrine control, neuronal function or neurological disorders, and the immune system, respectively. Then, the disease‐specific subnetwork was constructed and a number of novel genes potentially involved in MDD were identified. When more candidate genes associated with MDD are identified, the procedure outlined in this study should provide more detailed gene interaction and pathological molecular network on MDD. In addition, information on MDD from other sources can also be integrated into the framework used in this study; then, we will be able to obtain a more comprehensive and meaningful understanding on the molecular mechanisms on the pathogenesis of MDD.

## CONFLICT OF INTEREST

None declared.

## Data Availability

Supporting data are provided as additional supporting files.
