# Supplementary material for: Analyzing the genes and pathways related to major depressive disorder via a systems biology approach
Source: Brain Behav. 2019 Dec 25;10(2):e01502. doi: 10.1002/brb3.1502 (PMC7010578; doi:10.1002/brb3.1502)
Supplement: Supplementary file 1 [file BRB3-10-e01502-s001.doc]

**Supplemental Table S1**. List of genes associated with major depressive disorder

| **Gene symbol** | **Gene name** | **Reference (PMIDs)^a^** |
| --- | --- | --- |
| ABCB1 | ATP binding cassette subfamily B member 1 | 27439447; 27309038; 27565994; 25980509; 25847751; 25815420; 24200053; 22641028; 22306099; 19844206; 17913323; 23188198 |
| ABCB6 | ATP binding cassette subfamily B member 6 (Langereis blood group) | 19107115 |
| ACE | angiotensin I converting enzyme | 22036796; 19713413 |
| ACSM1 | acyl-CoA synthetase medium chain family member 1 | 25656805 |
| ACYP2 | acylphosphatase 2 | 25287955 |
| ADAM19 | ADAM metallopeptidase domain 19 | 25287955 |
| ADCY3 | adenylate cyclase 3 | 21042317 |
| ADCY6 | adenylate cyclase 6 | 20656788 |
| ADCY9 | adenylate cyclase 9 | 26745768; 19107115 |
| ADK | adenosine kinase | 26566975 |
| AKT1 | AKT serine/threonine kinase 1 | 27565994 |
| ANKZF1 | ankyrin repeat and zinc finger domain containing 1 | 19107115 |
| APAF1 | apoptotic peptidase activating factor 1 | 16231040;19455599 |
| APOE | apolipoprotein E | 26324127; 19135213; 17938638; 25287955 |
| ARNTL | aryl hydrocarbon receptor nuclear translocator like | 26566975 |
| ARNTL2 | aryl hydrocarbon receptor nuclear translocator like 2 | 26566975 |
| ARRB1 | arrestin beta 1 | 25294870 |
| AS3MT | arsenite methyltransferase | 28035465; 23453885 |
| ATF3 | activating transcription factor 3 | 25287955 |
| ATG7 | autophagy related 7 | 19107115 |
| ATG9A | autophagy related 9A | 19107115 |
| ATP6V1B2 | ATPase H+ transporting V1 subunit B2 | 25287955 |
| AVPR1B | arginine vasopressin receptor 1B | 25448875 |
| BBOX1 | gamma-butyrobetaine hydroxylase 1 | 25287955 |
| BCR | BCR, RhoGEF and GTPase activating protein | 26566975 |
| BDNF | brain derived neurotrophic factor | 27994504; 27787964; 27723767; 27439447; 26976307; 26960194; 26921055; 26717541; 27565994; 25980509; 25798331; 25745134; 25510949; 25106037; 25044977; 24607934; 23733090; 23733030; 23570888; 22965830; 22883353; 22681170; 22449891; 22194899; 21692988; 21420735; 20167454; 20033742; 19699537; 19376528; 22247094; 20934189; 19589373; 21129438; 21103954; 20674983;  20653908;20703451 |
| C3orf70 | chromosome 3 open reading frame 70 | 27519822 |
| CACNA1A | calcium voltage-gated channel subunit alpha1 A | 27439447; 27565994; 25287955;27091189 |
| CACNA1C | calcium voltage-gated channel subunit alpha1 C | 27439447; 27091189; 27565994; 24988482; 24612926; 24411473; 24262814; 23453885; 21042317; 19388002 |
| CACNA1D | calcium voltage-gated channel subunit alpha1 D | 23453885 |
| CACNA1E | calcium voltage-gated channel subunit alpha1 E | 23453885 |
| CACNA1S | calcium voltage-gated channel subunit alpha1 S | 23453885 |
| CACNA2D2 | calcium voltage-gated channel auxiliary subunit alpha2delta 2 | 23453885 |
| CACNA2D4 | calcium voltage-gated channel auxiliary subunit alpha2delta 4 | 23453885 |
| CACNB2 | calcium voltage-gated channel auxiliary subunit beta 2 | 26798408; 23453885;27091189 |
| CADPS | calcium dependent secretion activator | 25287955 |
| CAV2 | caveolin 2 | 26192912 |
| CCDC68 | coiled-coil domain containing 68 | 23453885 |
| CDC42SE2 | CDC42 small effector 2 | 26566975 |
| CHRFAM7A | CHRNA7 (exons 5-10) and FAM7A (exons A-E) fusion | 26566975 |
| CHST11 | carbohydrate sulfotransferase 11 | 25287955 |
| CMYA5 | cardiomyopathy associated 5 | 24988482 |
| CNIH4 | cornichon family AMPA receptor auxiliary protein 4 | 25287955 |
| CNNM2 | cyclin and CBS domain divalent metal cation transport mediator 2 | 23453885 |
| CNR1 | cannabinoid receptor 1 | 26566975; 26331953; 27565994; 23407780 |
| COMT | catechol-O-methyltransferase | 27723767; 26960194; 26813412; 26566975; 26021967; 25766270; 24751310; 23787408; 23618651; 23008195; 22483292; 21788083; 20071037; 19593178; 19095219; 19095219; 20627703 |
| CPXM2 | carboxypeptidase X, M14 family member 2 | 26655122 |
| CREB1 | cAMP responsive element binding protein 1 | 28225778; 26566975; 25755794; 25059218; 24955721; 24006268; 22293360; 19844206; 17300755; 21598377 |
| CRHR1 | corticotropin releasing hormone receptor 1 | 27549215; 27439447; 27565994; 25650683; 25448875; 23962971; 23529111; 22194899; 17258395; 22467522;18250257 |
| CRHR2 | corticotropin releasing hormone receptor 2 | 27549215; 22467522 |
| CRP | C-reactive protein | 25798331; 25603415 |
| CRTC1 | CREB regulated transcription coactivator 1 | 27002284 |
| CRY1 | cryptochrome circadian regulator 1 | 20072116; 24581835 |
| CTLA4 | cytotoxic T-lymphocyte associated protein 4 | 26566975; 21040781 |
| CYP2B6 | cytochrome P450 family 2 subfamily B member 6 | 26830411 |
| CYP2C19 | cytochrome P450 family 2 subfamily C member 19 | 26343256;25287955 |
| CYP2D6 | cytochrome P450 family 2 subfamily D member 6 | 23734807;25287955 |
| DAOA | D-amino acid oxidase activator | 26566975 |
| DIO1 | iodothyronine deiodinase 1 | 21563302 |
| DISC1 | disrupted in schizophrenia 1 | 25043320 |
| DNAJB2 | DnaJ heat shock protein family (Hsp40) member B2 | 25287955; 19107115 |
| DNPEP | aspartyl aminopeptidase | 19107115 |
| DOCK6 | dedicator of cytokinesis 6 | 25287955 |
| DRD1 | dopamine receptor D1 | 27565994; 26324127; 20558238 |
| DRD4 | dopamine receptor D4 | 26324127; 20558238; 17938638 |
| EGF | epidermal growth factor | 27755861 |
| EHD3 | EH domain containing 3 | 25287955; 22337703; 19107115 |
| EHHADH | enoyl-CoA hydratase and 3-hydroxyacyl CoA dehydrogenase | 25287955 |
| EIF2AK2 | eukaryotic translation initiation factor 2 alpha kinase 2 | 27151072 |
| ESR1 | estrogen receptor 1 | 22051074 |
| FANCI | Fanconi anemia complementation group I | 26798408 |
| FHOD1 | formin homology 2 domain containing 1 | 25287955 |
| FIGN | fidgetin, microtubule severing factor | 21621269 |
| FKBP4 | FK506 binding protein 4 | 20726698 |
| FKBP5 | FK506 binding protein 5 | 27549215; 26645208; 26076833; 25980509; 25448875; 25191701; 23406438; 20726698;  18191112;19199039 |
| FoxP2 | forkhead box P2 | 22404659 |
| FREM3 | FRAS1 related extracellular matrix 3 | 22337703; 25287955 |
| FTO | alpha-ketoglutarate dependent dioxygenase | 26122587; 25656805 |
| GABBR2 | gamma-aminobutyric acid type B receptor subunit 2 | 26892569 |
| GABRA4 | gamma-aminobutyric acid type A receptor alpha4 subunit | 26892569 |
| GABRB3 | gamma-aminobutyric acid type A receptor beta3 subunit | 26892569 |
| GABRD | gamma-aminobutyric acid type A receptor delta subunit | 26892569 |
| GABRG2 | gamma-aminobutyric acid type A receptor gamma2 subunit | 26892569 |
| GAL | galanin and GMAP prepropeptide | 21042317 |
| GHRHR | growth hormone releasing hormone receptor | 22449891 |
| GLB1L | galactosidase beta 1 like | 19107115 |
| GLT8D1 | glycosyltransferase 8 domain containing 1 | 28035465 |
| GNB1 | G protein subunit beta 1 | 27439447; 27565994 |
| GNB3 | G protein subunit beta 3 | 28225778; 26745768; 26324127; 25980509; 25451402; 25287955; 19560507; 17938638; 14647404 |
| GNL3 | G protein nucleolar 3 | 28035465 |
| GPX5 | glutathione peroxidase 5 | 28035465 |
| GRIA1 | glutamate ionotropic receptor AMPA type subunit 1 | 22057216 |
| GRIA2 | glutamate ionotropic receptor AMPA type subunit 2 | 22057216; 26892569 |
| GRIA4 | glutamate ionotropic receptor AMPA type subunit 4 | 22057216 |
| GRIK1 | glutamate ionotropic receptor kainate type subunit 1 | 26892569 |
| GRIK4 | glutamate ionotropic receptor kainate type subunit 4 | 17671280;23394390 |
| GRIN2A | glutamate ionotropic receptor NMDA type subunit 2A | 26892569 |
| GRIN2B | glutamate ionotropic receptor NMDA type subunit 2B | 24114429 |
| GRM7 | glutamate metabotropic receptor 7 | 26655190; 20038947; 21572167; 25287955 |
| GRM8 | glutamate metabotropic receptor 8 | 26655190; 25287955 |
| GSK3B | glycogen synthase kinase 3 beta | 20033742; 18195729; 20219685; 19581563;  20618448 |
| GYPE | glycophorin E (MNS blood group) | 19107115 |
| HCRTR1 | hypocretin receptor 1 | 26566975 |
| HOMER1 | homer scaffold protein 1 | 27964944 |
| HSD3B1 | hydroxy-delta-5-steroid dehydrogenase, 3 beta- and steroid delta-isomerase 1 | 22449891 |
| HTR1A | 5-hydroxytryptamine receptor 1A | 27756686; 26780922; 26561939; 18561871; 14507979; 19105200; 18706198; 20453658; 27439447; 27565994; 22752684; 19730445;  19326813;19437301;15691525;19813033 |
| HTR1B | 5-hydroxytryptamine receptor 1B | 26745768; 22907730 |
| HTR2A | 5-hydroxytryptamine receptor 2A | 27756686; 21917318; 26764241; 26745768; 24533444; 19590397; 20453658; 16127283; 14730199; 27439447; 26798408; 26764241; 27565994; 25980509; 25108775; 21838737; 17671280; 19560507 |
| HTR2C | 5-hydroxytryptamine receptor 2C | 26566975 |
| HTR4 | 5-hydroxytryptamine receptor 4 | 25522384 |
| HTR6 | 5-hydroxytryptamine receptor 6 | 15823158 |
| IDO1 | indoleamine 2,3-dioxygenase 1 | 22282879 |
| IDO2 | indoleamine 2,3-dioxygenase 2 | 22282879 |
| IGFBP3 | insulin like growth factor binding protein 3 | 27755861 |
| IKBKE | inhibitor of nuclear factor kappa B kinase subunit epsilon | 25798331; 19786281 |
| IL10 | interleukin 10 | 19786281 |
| IL1B | interleukin 1 beta | 25798331; 20044070; 26663052 |
| IL33 | interleukin 33 | 27054346 |
| IL6 | interleukin 6 | 27796193; 25466434 |
| ITIH1 | inter-alpha-trypsin inhibitor heavy chain 1 | 28035465 |
| ITIH3 | inter-alpha-trypsin inhibitor heavy chain 3 | 28035465; 23453885 |
| ITIH4 | inter-alpha-trypsin inhibitor heavy chain family member 4 | 28035465 |
| ITPR1 | inositol 1,4,5-trisphosphate receptor type 1 | 25287955 |
| KCNK2 | potassium two pore domain channel subfamily K member 2 | 26566975; 25535009 |
| KLHL29 | kelch like family member 29 | 25287955 |
| LAMC2 | laminin subunit gamma 2 | 19107115 |
| LHFPL2 | LHFPL tetraspan subfamily member 2 | 25287955 |
| LHPP | phospholysine phosphohistidine inorganic pyrophosphate phosphatase | 26176920; 26655122 |
| LRP5 | LDL receptor related protein 5 | 28035465 |
| LSAMP | limbic system-associated membrane protein | 22892717 |
| MAGI1 | membrane associated guanylate kinase, WW and PDZ domain containing 1 | 25993607 |
| MAOA | monoamine oxidase A | 27723767; 26566975; 24809685; 21605465; 20691428; 20175604; 20078943; 19859025; 25287955 |
| MAP3K13 | mitogen-activated protein kinase kinase kinase 13 | 27519822 |
| MCTP2 | multiple C2 and transmembrane domain containing 2 | 18367154 |
| MKLN1 | muskelin 1 | 26192912; 25287955 |
| MORC1 | MORC family CW-type zinc finger 1 | 25158004 |
| MTHFR | methylenetetrahydrofolate reductase | 27035272; 26960194; 26566975; 26324127; 26021967; 24532086; 24123968; 17938638; 24751310; 16223965 |
| MX1 | MX dynamin like GTPase 1 | 27151072 |
| MYBPC3 | myosin binding protein C, cardiac | 19107115 |
| MYO3A | myosin IIIA | 26566975 |
| MYT1L | myelin transcription factor 1 like | 26566975 |
| NAMPT | nicotinamide phosphoribosyltransferase | 26192912 |
| NBEAL2 | neurobeachin like 2 | 26798408 |
| NCAN | neurocan | 25815420;25801500 |
| NDUFV2 | NADH:ubiquinone oxidoreductase core subunit V2 | 26544616 |
| NEK4 | NIMA related kinase 4 | 28035465 |
| NFE2L3 | nuclear factor, erythroid 2 like 3 | 19107115 |
| NFKB1 | nuclear factor kappa B subunit 1 | 19107115 |
| NGF | nerve growth factor | 26021968 |
| NLGN1 | neuroligin 1 | 25287955 |
| NMNAT2 | nicotinamide nucleotide adenylyltransferase 2 | 19107115 |
| NPAS2 | neuronal PAS domain protein 2 | 20072116 |
| NPR3 | natriuretic peptide receptor 3 | 20072116 |
| NPY | neuropeptide Y | 23468908 |
| NR1D1 | nuclear receptor subfamily 1 group D member 1 | 26566975 |
| NR3C1 | nuclear receptor subfamily 3 group C member 1 | 27549215; 26782558; 25448875; 21764460 |
| NR3C2 | nuclear receptor subfamily 3 group C member 2 | 27549215 |
| NRG1 | neuregulin 1 | 26888291 |
| NRGN | neurogranin | 26828755 |
| NT5C2 | 5'-nucleotidase, cytosolic II | 23453885 |
| NTRK2 | neurotrophic receptor tyrosine kinase 2 | 19844206 |
| NTRK3 | neurotrophic receptor tyrosine kinase 3 | 18367154 |
| NVL | nuclear VCP-like | 25287955 |
| OPRM1 | polybromo 1 | 24766998 |
| OR4B1 | olfactory receptor family 4 subfamily B member 1 | 21621269 |
| P2RX7 | purinergic receptor P2X 7 | 23269209; 21438144; 16822851 |
| PARK2 | parkin RBR E3 ubiquitin protein ligase | 25287955; 21438144; 16822851; 23269209 |
| PARP14 | poly(ADP-ribose) polymerase family member 14 | 27151072 |
| PAWR | pro-apoptotic WT1 regulator | 20735158 |
| PBRM1 | polybromo 1 | 28035465 |
| PCLO | piccolo presynaptic cytomatrix protein | 26566975; 23620758; 22832909; 22386049; 22293360; 21621269; 19546850; 25287955;  24278217 |
| PDE11A | phosphodiesterase 11A | 26566975; 17008408 |
| PDE1C | phosphodiesterase 1C | 26192912 |
| PDE2A | phosphodiesterase 2A | 26566975 |
| PDE4B | phosphodiesterase 4B | 26566975; 18785206 |
| PDE5A | phosphodiesterase 5A | 26566975 |
| PDE6C | phosphodiesterase 6C | 22449891 |
| PDLIM5 | PDZ and LIM domain 5 | 22449891;18197271 |
| PGBD1 | piggyBac transposable element derived 1 | 28035465 |
| PHACTR3 | phosphatase and actin regulator 3 | 25287955 |
| PLD1 | phospholipase D1 | 26566975 |
| POMC | proopiomelanocortin | 26566975; 25448875 |
| PON1 | paraoxonase 1 | 24679385 |
| PRKAG2 | protein kinase AMP-activated non-catalytic subunit gamma 2 | 26192912 |
| PRKCG | protein kinase C gamma | 26921055 |
| PROKR2 | prokineticin receptor 2 | 26566975 |
| PSMB4 | proteasome subunit beta 4 | 26566975; 18504423 |
| PSMC1 | proteasome 26S subunit, ATPase 1 | 27151072 |
| PSMC3 | proteasome 26S subunit, ATPase 3 | 19107115 |
| PSMD9 | proteasome 26S subunit, non-ATPase 9 | 22449891 |
| PTPRN | protein tyrosine phosphatase, receptor type N | 19107115 |
| PTPRR | protein tyrosine phosphatase, receptor type R | 22100128; 25287955 |
| RAPSN | receptor associated protein of the synapse | 19107115 |
| RGMA | repulsive guidance molecule BMP co-receptor a | 18367154 |
| RHCG | Rh family C glycoprotein | 18367154 |
| RNF123 | ring finger protein 123 | 21982424 |
| RPP21 | ribonuclease P/MRP subunit p21 | 28035465 |
| RPS6KB1 | ribosomal protein S6 kinase B1 | 27565994 |
| SEMA5A | semaphorin 5A | 25287955 |
| SHC3 | SHC adaptor protein 3 | 25287955 |
| SIGMAR1 | sigma non-opioid intracellular receptor 1 | 28144920; 20178807 |
| SIRT1 | sirtuin 1 | 26566975; 20451257; 26176920 |
| SLC1A2 | solute carrier family 1 member 2 | 26892569 |
| SLC1A4 | solute carrier family 1 member 4 | 25287955 |
| SLC25A21 | solute carrier family 25 member 21 | 25287955 |
| SLC29A3 | solute carrier family 29 member 3 | 26566975 |
| SLC36A1 | solute carrier family 36 member 1 | 25287955 |
| SLC39A13 | solute carrier family 39 member 13 | 19107115 |
| SLC6A1 | solute carrier family 6 member 1 | 26892569 |
| SLC6A15 | solute carrier family 6 member 15 | 27723767; 21521612 |
| SLC6A2 | solute carrier family 6 member 2 | 26566975; 25512257; 20351714; 26960194; 19844206; 19105200; 25739526; 19564048; 18779921 |
| SLC6A3 | solute carrier family 6 member 3 | 26324127; 20391341; 19844206; 17938638 |
| SLC6A4 | solute carrier family 6 member 4 | 26976307; 26674707; 23787408; 21692988; 27723767; 27439447; 25827644; 23969989; 23618376; 23021380; 22921522; 21862951; 21420735; 19125107; 18801474; 18615541; 17618721; 16319503; 27439447; 25739526; 25510949; 17853254; 27964944; 27023264; 26745768; 26324127; 27565994;24657235;  19437301;15691525;18486105 |
| SLCO3A1 | solute carrier organic anion transporter family member 3A1 | 18367154 |
| SMG7 | SMG7, nonsense mediated mRNA decay factor | 19107115 |
| SMIM4 | small integral membrane protein 4 | 28035465 |
| SNAP25 | synaptosome associated protein 25 | 28035465 |
| SNORD19 | small nucleolar RNA, C/D box 19 | 28035465 |
| SNORD69 | small nucleolar RNA, C/D box 69 | 28035465 |
| SP4 | Sp4 transcription factor | 25287955 |
| SPI1 | Spi-1 proto-oncogene | 19107115 |
| STAT1 | signal transducer and activator of transcription 1 | 27151072 |
| STK16 | serine/threonine kinase 16 | 19107115 |
| SV2B | synaptic vesicle glycoprotein 2B | 18367154 |
| SYPL1 | synaptophysin like 1 | 26192912 |
| TAL1 | TAL bHLH transcription factor 1, erythroid differentiation factor | 26717541 |
| TBC1D21 | TBC1 domain family member 21 | 25287955 |
| TBX21 | T-box 21 | 18504423 |
| TEF | TEF, PAR bZIP transcription factor | 27964944; 24581835 |
| TGFB1 | transforming growth factor beta 1 | 25466434 |
| TNF | tumor necrosis factor | 25798331; 20351714; 19618378 |
| TP53 | tumor protein p53 | 27755861 |
| TPH1 | tryptophan hydroxylase 1 | 27439447; 27565994; 23597148; 23221997; 22697203; 20945066; 19874868; 19590397; 19500158; 16165107 |
| TPH2 | tryptophan hydroxylase 2 | 27723767; 26745768; 25798331; 23063133; 19272410; 15124006; 26386440; 25287955; 26386440 |
| TRIM10 | tripartite motif containing 10 | 28035465 |
| TRIM26 | tripartite motif containing 26 | 28035465 |
| TRIM39 | tripartite motif containing 39 | 28035465 |
| TSNAX | translin associated factor X | 26566975; 25043320; 19760522 |
| UGT2A1 | UDP glucuronosyltransferase family 2 member A1 complex locus | 25287955; 19107115 |
| UGT2A2 | UDP glucuronosyltransferase family 2 member A2 | 19107115 |
| UGT2B4 | UDP glucuronosyltransferase family 2 member B4 | 25287955 |
| UNC13A | unc-13 homolog A | 25287955 |
| VCAN | versican | 25287955 |
| VGLL2 | vestigial like family member 2 | 19107115 |
| VGLL4 | vestigial like family member 4 | 25287955; 19107115 |
| VIPR2 | vasoactive intestinal peptide receptor 2 | 20072116 |
| VPS33B | VPS33B, late endosome and lysosome associated | 18367154 |
| VPS8 | VPS8, CORVET complex subunit | 27519822 |
| WDR26 | WD repeat domain 26 | 25287955 |
| WDR82 | WD repeat domain 82 | 27151072 |
| XDH | xanthine dehydrogenase | 19107115 |
| YY1 | YY1 transcription factor | 27151072 |
| ZNF34 | zinc finger protein 34 | 26823146 |
| ZNF804A | zinc finger protein 804A | 25162540 |
| ZSCAN12 | zinc finger and SCAN domain containing 12 | 28035465 |

^a^ References are designated by PMIDs, which are the identifiers of PubMed.

**Supplement Table 2**. Terms of Gene Ontology Biological Process siginficantly enriched in MDD-related genes

| **GO ID** | **GO term** | **p-value** | **FDR^a^** |
| --- | --- | --- | --- |
| GO:0007267 | cell-cell signaling | 2.90×10^-39^ | 1.56×10^-35^ |
| GO:0099536 | synaptic signaling | 6.44×10^-36^ | 1.73×10^-32^ |
| GO:0099537 | trans-synaptic signaling | 2.88×10^-35^ | 3.09×10^-32^ |
| GO:0007610 | behavior | 1.12×10^-30^ | 1.00×10^-27^ |
| GO:0071705 | nitrogen compound transport | 9.32×10^-24^ | 6.25×10^-21^ |
| GO:0046903 | secretion | 2.44×10^-22^ | 1.33×10^-19^ |
| GO:0023061 | signal release | 9.29×10^-22^ | 4.16×10^-19^ |
| GO:0051049 | regulation of transport | 2.12×10^-20^ | 8.75×10^-18^ |
| GO:0006811 | ion transport | 2.40×10^-20^ | 9.19×10^-18^ |
| GO:0010817 | regulation of hormone levels | 2.83×10^-19^ | 8.29×10^-17^ |
| GO:0032940 | secretion by cell | 2.94×10^-19^ | 8.29×10^-17^ |
| GO:1901698 | response to nitrogen compound | 3.19×10^-19^ | 8.56×10^-17^ |
| GO:0007270 | neuron-neuron synaptic transmission | 6.36×10^-19^ | 1.62×10^-16^ |
| GO:0003013 | circulatory system process | 3.66×10^-18^ | 7.56×10^-16^ |
| GO:1901700 | response to oxygen-containing compound | 5.11×10^-18^ | 1.01×10^-15^ |
| GO:0009914 | hormone transport | 5.84×10^-18^ | 1.12×10^-15^ |
| GO:0050806 | positive regulation of synaptic transmission | 8.09×10^-18^ | 1.50×10^-15^ |
| GO:0046883 | regulation of hormone secretion | 1.05×10^-17^ | 1.88×10^-15^ |
| GO:0046879 | hormone secretion | 1.67×10^-17^ | 2.80×10^-15^ |
| GO:0050804 | modulation of synaptic transmission | 2.52×10^-17^ | 1.06×10^-15^ |
| GO:0048511 | rhythmic process | 3.46×10^-17^ | 5.62×10^-15^ |
| GO:0055085 | transmembrane transport | 8.22×10^-17^ | 1.30×10^-14^ |
| GO:0060341 | regulation of cellular localization | 1.50×10^-16^ | 2.24×10^-14^ |
| GO:0009719 | response to endogenous stimulus | 2.48×10^-16^ | 3.60×10^-14^ |
| GO:0043269 | regulation of ion transport | 2.98×10^-16^ | 4.10×10^-14^ |
| GO:0007613 | memory | 3.44×10^-16^ | 4.62×10^-14^ |
| GO:0030072 | peptide hormone secretion | 1.25×10^-15^ | 1.63×10^-13^ |
| GO:0002790 | peptide secretion | 2.51×10^-15^ | 3.04×10^-13^ |
| GO:0050803 | regulation of synapse structure or activity | 4.50×10^-15^ | 5.25×10^-13^ |
| GO:0006812 | cation transport | 6.28×10^-15^ | 6.87×10^-13^ |
| GO:0035249 | synaptic transmission, glutamatergic | 1.60×10^-14^ | 1.65×10^-12^ |
| GO:0031328 | positive regulation of cellular biosynthetic process | 3.43×10^-14^ | 3.47×10^-12^ |
| GO:0045935 | positive regulation of nucleobase-containing compound metabolic process | 4.04×10^-14^ | 4.01×10^-12^ |
| GO:0030073 | insulin secretion | 7.70×10^-14^ | 7.01×10^-12^ |
| GO:0009891 | positive regulation of biosynthetic process | 8.84×10^-14^ | 7.90×10^-12^ |
| GO:0033555 | multicellular organismal response to stress | 1.04×10^-13^ | 9.18×10^-12^ |
| GO:0006140 | regulation of nucleotide metabolic process | 1.11×10^-13^ | 9.47×10^-12^ |
| GO:0072511 | divalent inorganic cation transport | 1.28×10^-13^ | 1.07×10^-11^ |
| GO:0006163 | purine nucleotide metabolic process | 1.35×10^-13^ | 1.12×10^-11^ |
| GO:0051051 | negative regulation of transport | 1.56×10^-13^ | 1.27×10^-11^ |
| GO:1901699 | cellular response to nitrogen compound | 2.07×10^-13^ | 1.61×10^-11^ |
| GO:0006820 | anion transport | 3.24×10^-13^ | 2.45×10^-11^ |
| GO:0033993 | response to lipid | 3.66×10^-13^ | 2.73×10^-11^ |
| GO:0071495 | cellular response to endogenous stimulus | 4.03×10^-13^ | 2.96×10^-11^ |
| GO:0015696 | ammonium transport | 4.17×10^-13^ | 3.03×10^-11^ |
| GO:0050877 | neurological system process | 5.66×10^-13^ | 1.23×10^-11^ |
| GO:0071417 | cellular response to organonitrogen compound | 5.72×10^-13^ | 4.07×10^-11^ |
| GO:0009259 | ribonucleotide metabolic process | 5.98×10^-13^ | 4.07×10^-11^ |
| GO:0007612 | learning | 6.46×10^-13^ | 4.33×10^-11^ |
| GO:0007187 | G-protein coupled receptor signaling pathway, coupled to cyclic nucleotide second messenger | 6.65×10^-13^ | 4.41×10^-11^ |
| GO:0051966 | regulation of synaptic transmission, glutamatergic | 7.87×10^-13^ | 5.15×10^-11^ |
| GO:0051048 | negative regulation of secretion | 8.83×10^-13^ | 5.71×10^-11^ |
| GO:0090276 | regulation of peptide hormone secretion | 9.63×10^-13^ | 6.15×10^-11^ |
| GO:0006753 | nucleoside phosphate metabolic process | 9.95×10^-13^ | 6.28×10^-11^ |
| GO:0006836 | neurotransmitter transport | 1.08×10^-12^ | 6.72×10^-11^ |
| GO:0019693 | ribose phosphate metabolic process | 1.25×10^-12^ | 7.72×10^-11^ |
| GO:0048167 | regulation of synaptic plasticity | 1.39×10^-12^ | 8.48×10^-11^ |
| GO:0002791 | regulation of peptide secretion | 1.47×10^-12^ | 8.89×10^-11^ |
| GO:1900371 | regulation of purine nucleotide biosynthetic process | 1.79×10^-12^ | 1.07×10^-10^ |
| GO:1901293 | nucleoside phosphate biosynthetic process | 1.99×10^-12^ | 1.16×10^-10^ |
| GO:0030808 | regulation of nucleotide biosynthetic process | 2.01×10^-12^ | 1.16×10^-10^ |
| GO:0009306 | protein secretion | 2.03×10^-12^ | 1.16×10^-10^ |
| GO:0050708 | regulation of protein secretion | 2.03×10^-12^ | 1.16×10^-10^ |
| GO:0009725 | response to hormone | 2.06×10^-12^ | 1.16×10^-10^ |
| GO:0050796 | regulation of insulin secretion | 2.40×10^-12^ | 1.34×10^-10^ |
| GO:0042493 | response to drug | 2.65×10^-12^ | 1.47×10^-10^ |
| GO:0060291 | long-term synaptic potentiation | 2.98×10^-12^ | 1.63×10^-10^ |
| GO:0009117 | nucleotide metabolic process | 3.05×10^-12^ | 1.65×10^-10^ |
| GO:0019233 | sensory perception of pain | 3.42×10^-12^ | 1.83×10^-10^ |
| GO:0010942 | positive regulation of cell death | 3.45×10^-12^ | 1.84×10^-10^ |
| GO:0052652 | cyclic purine nucleotide metabolic process | 3.53×10^-12^ | 1.86×10^-10^ |
| GO:0046942 | carboxylic acid transport | 3.64×10^-12^ | 1.90×10^-10^ |
| GO:0009190 | cyclic nucleotide biosynthetic process | 3.94×10^-12^ | 1.99×10^-10^ |
| GO:0015849 | organic acid transport | 4.74×10^-12^ | 2.37×10^-10^ |
| GO:0009152 | purine ribonucleotide biosynthetic process | 4.97×10^-12^ | 2.47×10^-10^ |
| GO:0030802 | regulation of cyclic nucleotide biosynthetic process | 6.98×10^-12^ | 3.43×10^-10^ |
| GO:0030817 | regulation of cAMP biosynthetic process | 7.02×10^-12^ | 3.43×10^-10^ |
| GO:0006164 | purine nucleotide biosynthetic process | 7.90×10^-12^ | 3.82×10^-10^ |
| GO:0090087 | regulation of peptide transport | 8.46×10^-12^ | 4.05×10^-10^ |
| GO:0015850 | organic hydroxy compound transport | 8.52×10^-12^ | 4.05×10^-10^ |
| GO:1901701 | cellular response to oxygen-containing compound | 1.09×10^-11^ | 5.12×10^-10^ |
| GO:0009165 | nucleotide biosynthetic process | 1.10×10^-11^ | 5.12×10^-10^ |
| GO:0001505 | regulation of neurotransmitter levels | 1.12×10^-11^ | 5.16×10^-10^ |
| GO:0006865 | amino acid transport | 1.13×10^-11^ | 5.19×10^-10^ |
| GO:0015844 | monoamine transport | 1.14×10^-11^ | 5.19×10^-10^ |
| GO:0046717 | acid secretion | 1.32×10^-11^ | 5.95×10^-10^ |
| GO:0009260 | ribonucleotide biosynthetic process | 1.33×10^-11^ | 5.96×10^-10^ |
| GO:0030799 | regulation of cyclic nucleotide metabolic process | 1.38×10^-11^ | 6.14×10^-10^ |
| GO:0006171 | cAMP biosynthetic process | 1.49×10^-11^ | 6.57×10^-10^ |
| GO:0070588 | calcium ion transmembrane transport | 1.66×10^-11^ | 7.24×10^-10^ |
| GO:0046390 | ribose phosphate biosynthetic process | 1.79×10^-11^ | 7.72×10^-10^ |
| GO:0072522 | purine-containing compound biosynthetic process | 1.92×10^-11^ | 8.17×10^-10^ |
| GO:0045944 | positive regulation of transcription from RNA polymerase II promoter | 2.00×10^-11^ | 3.54×10^-10^ |
| GO:0042391 | regulation of membrane potential | 2.04×10^-11^ | 8.60×10^-10^ |
| GO:0003018 | vascular process in circulatory system | 3.32×10^-11^ | 1.39×10^-9^ |
| GO:0032870 | cellular response to hormone stimulus | 3.40×10^-11^ | 1.41×10^-9^ |
| GO:0098660 | inorganic ion transmembrane transport | 3.52×10^-11^ | 1.45×10^-9^ |
| GO:0007631 | feeding behavior | 3.83×10^-11^ | 1.57×10^-9^ |
| GO:0042752 | regulation of circadian rhythm | 3.96×10^-11^ | 1.61×10^-9^ |
| GO:0043279 | response to alkaloid | 3.99×10^-11^ | 1.61×10^-9^ |
| GO:0019932 | second-messenger-mediated signaling | 4.32×10^-11^ | 1.73×10^-9^ |
| GO:0043065 | positive regulation of apoptotic process | 4.92×10^-11^ | 1.96×10^-9^ |
| GO:1903531 | negative regulation of secretion by cell | 5.09×10^-11^ | 2.01×10^-9^ |
| GO:0010628 | positive regulation of gene expression | 5.27×10^-11^ | 2.06×10^-9^ |
| GO:0007626 | locomotory behavior | 5.69×10^-11^ | 2.21×10^-9^ |
| GO:1901652 | response to peptide | 6.19×10^-11^ | 2.39×10^-9^ |
| GO:0043068 | positive regulation of programmed cell death | 6.26×10^-11^ | 2.40×10^-9^ |
| GO:0030814 | regulation of cAMP metabolic process | 6.69×10^-11^ | 2.55×10^-9^ |
| GO:0051050 | positive regulation of transport | 7.05×10^-11^ | 2.66×10^-9^ |
| GO:0051955 | regulation of amino acid transport | 7.17×10^-11^ | 2.69×10^-9^ |
| GO:0030001 | metal ion transport | 7.67×10^-11^ | 2.86×10^-9^ |
| GO:0051047 | positive regulation of secretion | 8.52×10^-11^ | 3.15×10^-9^ |
| GO:1903508 | positive regulation of nucleic acid-templated transcription | 1.01×10^-10^ | 3.69×10^-9^ |
| GO:0045893 | positive regulation of transcription, DNA-templated | 1.01×10^-10^ | 3.69×10^-9^ |
| GO:0009628 | response to abiotic stimulus | 1.36×10^-10^ | 4.94×10^-9^ |
| GO:0030534 | adult behavior | 1.42×10^-10^ | 5.11×10^-9^ |
| GO:1902680 | positive regulation of RNA biosynthetic process | 1.55×10^-10^ | 5.51×10^-9^ |
| GO:0010941 | regulation of cell death | 1.84×10^-10^ | 6.45×10^-9^ |
| GO:0099531 | presynaptic process involved in chemical synaptic transmission | 1.87×10^-10^ | 6.51×10^-9^ |
| GO:0031401 | positive regulation of protein modification process | 1.99×10^-10^ | 6.88×10^-9^ |
| GO:0045761 | regulation of adenylate cyclase activity | 2.76×10^-10^ | 9.51×10^-9^ |
| GO:0043434 | response to peptide hormone | 2.99×10^-10^ | 1.02×10^-8^ |
| GO:0070509 | calcium ion import | 3.20×10^-10^ | 1.08×10^-8^ |
| GO:0007186 | G-protein coupled receptor signaling pathway | 3.22×10^-10^ | 1.08×10^-8^ |
| GO:0007215 | glutamate receptor signaling pathway | 3.25×10^-10^ | 1.08×10^-8^ |
| GO:0050808 | synapse organization | 3.27×10^-10^ | 1.08×10^-8^ |
| GO:0098655 | cation transmembrane transport | 3.36×10^-10^ | 1.11×10^-8^ |
| GO:1903532 | positive regulation of secretion by cell | 4.51×10^-10^ | 1.47×10^-8^ |
| GO:0051254 | positive regulation of RNA metabolic process | 4.59×10^-10^ | 1.48×10^-8^ |
| GO:0051241 | negative regulation of multicellular organismal process | 4.66×10^-10^ | 1.49×10^-8^ |
| GO:0023057 | negative regulation of signaling | 4.95×10^-10^ | 1.56×10^-8^ |
| GO:0034765 | regulation of ion transmembrane transport | 5.56×10^-10^ | 1.73×10^-8^ |
| GO:0023014 | signal transduction by protein phosphorylation | 6.11×10^-10^ | 1.90×10^-8^ |
| GO:0032270 | positive regulation of cellular protein metabolic process | 6.58×10^-10^ | 2.03×10^-8^ |
| GO:0051094 | positive regulation of developmental process | 6.67×10^-10^ | 2.04×10^-8^ |
| GO:0010648 | negative regulation of cell communication | 7.54×10^-10^ | 2.30×10^-8^ |
| GO:0000165 | MAPK cascade | 7.91×10^-10^ | 2.38×10^-8^ |
| GO:0071396 | cellular response to lipid | 8.63×10^-10^ | 2.59×10^-8^ |
| GO:1903522 | regulation of blood circulation | 9.24×10^-10^ | 2.75×10^-8^ |
| GO:0010557 | positive regulation of macromolecule biosynthetic process | 9.87×10^-10^ | 2.91×10^-8^ |
| GO:0048545 | response to steroid hormone | 1.05×10^-9^ | 3.07×10^-8^ |
| GO:0042417 | dopamine metabolic process | 1.10×10^-9^ | 3.17×10^-8^ |
| GO:0043271 | negative regulation of ion transport | 1.10×10^-9^ | 3.17×10^-8^ |
| GO:0007568 | aging | 1.22×10^-9^ | 3.49×10^-8^ |
| GO:0034762 | regulation of transmembrane transport | 1.27×10^-9^ | 3.62×10^-8^ |
| GO:1901135 | carbohydrate derivative metabolic process | 1.42×10^-9^ | 4.02×10^-8^ |
| GO:0030818 | negative regulation of cAMP biosynthetic process | 1.42×10^-9^ | 4.02×10^-8^ |
| GO:0045937 | positive regulation of phosphate metabolic process | 1.51×10^-9^ | 4.22×10^-8^ |
| GO:0010562 | positive regulation of phosphorus metabolic process | 1.51×10^-9^ | 4.22×10^-8^ |
| GO:0032890 | regulation of organic acid transport | 1.60×10^-9^ | 4.42×10^-8^ |
| GO:0098662 | inorganic cation transmembrane transport | 1.77×10^-9^ | 4.85×10^-8^ |
| GO:0031667 | response to nutrient levels | 1.89×10^-9^ | 5.13×10^-8^ |
| GO:0051240 | positive regulation of multicellular organismal process | 1.96×10^-9^ | 5.29×10^-8^ |
| GO:0031279 | regulation of cyclase activity | 2.19×10^-9^ | 5.88×10^-8^ |
| GO:0099565 | chemical synaptic transmission, postsynaptic | 2.24×10^-9^ | 5.97×10^-8^ |
| GO:0042592 | homeostatic process | 2.27×10^-9^ | 6.02×10^-8^ |
| GO:0015872 | dopamine transport | 2.33×10^-9^ | 6.13×10^-8^ |
| GO:0030803 | negative regulation of cyclic nucleotide biosynthetic process | 2.33×10^-9^ | 6.13×10^-8^ |
| GO:0010563 | negative regulation of phosphorus metabolic process | 2.47×10^-9^ | 6.44×10^-8^ |
| GO:0051402 | neuron apoptotic process | 2.62×10^-9^ | 6.80×10^-8^ |
| GO:0042220 | response to cocaine | 2.73×10^-9^ | 7.05×10^-8^ |
| GO:0051339 | regulation of lyase activity | 2.85×10^-9^ | 7.33×10^-8^ |
| GO:0042053 | regulation of dopamine metabolic process | 3.17×10^-9^ | 8.11×10^-8^ |
| GO:0006584 | catecholamine metabolic process | 3.29×10^-9^ | 8.32×10^-8^ |
| GO:0043523 | regulation of neuron apoptotic process | 3.34×10^-9^ | 8.37×10^-8^ |
| GO:0009967 | positive regulation of signal transduction | 3.39×10^-9^ | 8.47×10^-8^ |
| GO:0019935 | cyclic-nucleotide-mediated signaling | 3.50×10^-9^ | 8.65×10^-8^ |
| GO:0010876 | lipid localization | 3.53×10^-9^ | 8.69×10^-8^ |
| GO:2000026 | regulation of multicellular organismal development | 3.63×10^-9^ | 8.88×10^-8^ |
| GO:0008306 | associative learning | 3.69×10^-9^ | 8.96×10^-8^ |
| GO:0015711 | organic anion transport | 3.69×10^-9^ | 8.96×10^-8^ |
| GO:0030815 | negative regulation of cAMP metabolic process | 3.72×10^-9^ | 8.98×10^-8^ |
| GO:0006468 | protein phosphorylation | 4.16×10^-9^ | 1.00×10^-7^ |
| GO:0051223 | regulation of protein transport | 4.29×10^-9^ | 1.03×10^-7^ |
| GO:0001662 | behavioral fear response | 4.65×10^-9^ | 1.10×10^-7^ |
| GO:0030809 | negative regulation of nucleotide biosynthetic process | 4.65×10^-9^ | 1.10×10^-7^ |
| GO:1900372 | negative regulation of purine nucleotide biosynthetic process | 4.65×10^-9^ | 1.10×10^-7^ |
| GO:0042069 | regulation of catecholamine metabolic process | 4.83×10^-9^ | 1.13×10^-7^ |
| GO:1905114 | cell surface receptor signaling pathway involved in cell-cell signaling | 4.89×10^-9^ | 1.14×10^-7^ |
| GO:1901575 | organic substance catabolic process | 5.02×10^-9^ | 1.17×10^-7^ |
| GO:0019220 | regulation of phosphate metabolic process | 5.04×10^-9^ | 1.17×10^-7^ |
| GO:0051247 | positive regulation of protein metabolic process | 5.72×10^-9^ | 1.31×10^-7^ |
| GO:0002209 | behavioral defense response | 5.77×10^-9^ | 1.32×10^-7^ |
| GO:0009308 | amine metabolic process | 6.62×10^-9^ | 1.49×10^-7^ |
| GO:0030800 | negative regulation of cyclic nucleotide metabolic process | 7.13×10^-9^ | 1.59×10^-7^ |
| GO:0032880 | regulation of protein localization | 8.57×10^-9^ | 1.89×10^-7^ |
| GO:0014054 | positive regulation of gamma-aminobutyric acid secretion | 8.61×10^-9^ | 1.89×10^-7^ |
| GO:0051960 | regulation of nervous system development | 8.67×10^-9^ | 1.90×10^-7^ |
| GO:0019725 | cellular homeostasis | 9.73×10^-9^ | 2.12×10^-7^ |
| GO:0045598 | regulation of fat cell differentiation | 1.01×10^-8^ | 2.20×10^-7^ |
| GO:0051957 | positive regulation of amino acid transport | 1.04×10^-8^ | 2.24×10^-7^ |
| GO:0051235 | maintenance of location | 1.09×10^-8^ | 2.33×10^-7^ |
| GO:0010976 | positive regulation of neuron projection development | 1.18×10^-8^ | 2.50×10^-7^ |
| GO:0006835 | dicarboxylic acid transport | 1.22×10^-8^ | 2.57×10^-7^ |
| GO:0070201 | regulation of establishment of protein localization | 1.22×10^-8^ | 2.57×10^-7^ |
| GO:0045595 | regulation of cell differentiation | 1.22×10^-8^ | 2.57×10^-7^ |
| GO:0050795 | regulation of behavior | 1.24×10^-8^ | 2.60×10^-7^ |
| GO:0019637 | organophosphate metabolic process | 1.26×10^-8^ | 2.63×10^-7^ |
| GO:0046928 | regulation of neurotransmitter secretion | 1.27×10^-8^ | 2.65×10^-7^ |
| GO:0045471 | response to ethanol | 1.28×10^-8^ | 2.65×10^-7^ |
| GO:0010975 | regulation of neuron projection development | 1.30×10^-8^ | 2.68×10^-7^ |
| GO:0051932 | synaptic transmission, GABAergic | 1.30×10^-8^ | 2.68×10^-7^ |
| GO:0050880 | regulation of blood vessel size | 1.47×10^-8^ | 3.02×10^-7^ |
| GO:0035150 | regulation of tube size | 1.59×10^-8^ | 3.25×10^-7^ |
| GO:1901215 | negative regulation of neuron death | 1.72×10^-8^ | 3.49×10^-7^ |
| GO:0043408 | regulation of MAPK cascade | 1.72×10^-8^ | 3.49×10^-7^ |
| GO:0051248 | negative regulation of protein metabolic process | 1.99×10^-8^ | 3.99×10^-7^ |
| GO:1900543 | negative regulation of purine nucleotide metabolic process | 2.33×10^-8^ | 4.67×10^-7^ |
| GO:0031400 | negative regulation of protein modification process | 2.60×10^-8^ | 5.19×10^-7^ |
| GO:0043085 | positive regulation of catalytic activity | 2.75×10^-8^ | 5.44×10^-7^ |
| GO:0009611 | response to wounding | 2.76×10^-8^ | 5.44×10^-7^ |
| GO:0035235 | ionotropic glutamate receptor signaling pathway | 2.83×10^-8^ | 5.55×10^-7^ |
| GO:1901566 | organonitrogen compound biosynthetic process | 3.10×10^-8^ | 6.06×10^-7^ |
| GO:0045980 | negative regulation of nucleotide metabolic process | 3.11×10^-8^ | 6.06×10^-7^ |
| GO:0009416 | response to light stimulus | 3.19×10^-8^ | 6.20×10^-7^ |
| GO:0048589 | developmental growth | 3.27×10^-8^ | 6.33×10^-7^ |
| GO:0051924 | regulation of calcium ion transport | 3.33×10^-8^ | 6.42×10^-7^ |
| GO:0033002 | muscle cell proliferation | 3.60×10^-8^ | 6.92×10^-7^ |
| GO:0007214 | gamma-aminobutyric acid signaling pathway | 3.82×10^-8^ | 7.32×10^-7^ |
| GO:0050966 | detection of mechanical stimulus involved in sensory perception of pain | 3.94×10^-8^ | 7.48×10^-7^ |
| GO:0014051 | gamma-aminobutyric acid secretion | 3.94×10^-8^ | 7.48×10^-7^ |
| GO:1900273 | positive regulation of long-term synaptic potentiation | 3.94×10^-8^ | 7.48×10^-7^ |
| GO:0048265 | response to pain | 4.05×10^-8^ | 7.62×10^-7^ |
| GO:0032496 | response to lipopolysaccharide | 4.07×10^-8^ | 7.64×10^-7^ |
| GO:0007269 | neurotransmitter secretion | 4.39×10^-8^ | 8.15×10^-7^ |
| GO:0099643 | signal release from synapse | 4.39×10^-8^ | 8.15×10^-7^ |
| GO:0001508 | action potential | 4.49×10^-8^ | 8.31×10^-7^ |
| GO:0042430 | indole-containing compound metabolic process | 5.10×10^-8^ | 9.41×10^-7^ |
| GO:0042981 | regulation of apoptotic process | 5.17×10^-8^ | 9.50×10^-7^ |
| GO:0033238 | regulation of cellular amine metabolic process | 5.30×10^-8^ | 9.70×10^-7^ |
| GO:0043270 | positive regulation of ion transport | 5.92×10^-8^ | 1.08×10^-6^ |
| GO:0090407 | organophosphate biosynthetic process | 6.47×10^-8^ | 1.17×10^-6^ |
| GO:0051341 | regulation of oxidoreductase activity | 6.61×10^-8^ | 1.19×10^-6^ |
| GO:0001504 | neurotransmitter uptake | 6.73×10^-8^ | 1.21×10^-6^ |
| GO:0015718 | monocarboxylic acid transport | 6.92×10^-8^ | 1.24×10^-6^ |
| GO:0050805 | negative regulation of synaptic transmission | 6.97×10^-8^ | 1.24×10^-6^ |
| GO:0001934 | positive regulation of protein phosphorylation | 7.16×10^-8^ | 1.27×10^-6^ |
| GO:0043067 | regulation of programmed cell death | 7.23×10^-8^ | 1.28×10^-6^ |
| GO:0015672 | monovalent inorganic cation transport | 7.38×10^-8^ | 1.30×10^-6^ |
| GO:1903792 | negative regulation of anion transport | 7.50×10^-8^ | 1.32×10^-6^ |
| GO:0032892 | positive regulation of organic acid transport | 7.50×10^-8^ | 1.32×10^-6^ |
| GO:0045597 | positive regulation of cell differentiation | 8.55×10^-8^ | 1.49×10^-6^ |
| GO:0071375 | cellular response to peptide hormone stimulus | 8.61×10^-8^ | 1.50×10^-6^ |
| GO:0002237 | response to molecule of bacterial origin | 8.62×10^-8^ | 1.50×10^-6^ |
| GO:0033500 | carbohydrate homeostasis | 8.91×10^-8^ | 1.53×10^-6^ |
| GO:0042593 | glucose homeostasis | 8.91×10^-8^ | 1.53×10^-6^ |
| GO:0007210 | serotonin receptor signaling pathway | 8.94×10^-8^ | 1.53×10^-6^ |
| GO:0090066 | regulation of anatomical structure size | 9.17×10^-8^ | 1.57×10^-6^ |
| GO:0014052 | regulation of gamma-aminobutyric acid secretion | 9.99×10^-8^ | 1.70×10^-6^ |
| GO:0032269 | negative regulation of cellular protein metabolic process | 1.02×10^-7^ | 1.73×10^-6^ |
| GO:0006915 | apoptotic process | 1.10×10^-7^ | 1.85×10^-6^ |
| GO:0046434 | organophosphate catabolic process | 1.15×10^-7^ | 1.94×10^-6^ |
| GO:0032768 | regulation of monooxygenase activity | 1.16×10^-7^ | 1.95×10^-6^ |
| GO:0042310 | vasoconstriction | 1.24×10^-7^ | 2.09×10^-6^ |
| GO:0030162 | regulation of proteolysis | 1.28×10^-7^ | 2.14×10^-6^ |
| GO:0007189 | adenylate cyclase-activating G-protein coupled receptor signaling pathway | 1.29×10^-7^ | 2.16×10^-6^ |
| GO:0045773 | positive regulation of axon extension | 1.32×10^-7^ | 2.20×10^-6^ |
| GO:0048585 | negative regulation of response to stimulus | 1.36×10^-7^ | 2.26×10^-6^ |
| GO:0040008 | regulation of growth | 1.38×10^-7^ | 2.28×10^-6^ |
| GO:0051954 | positive regulation of amine transport | 1.58×10^-7^ | 2.60×10^-6^ |
| GO:0043410 | positive regulation of MAPK cascade | 1.67×10^-7^ | 2.72×10^-6^ |
| GO:0010959 | regulation of metal ion transport | 1.75×10^-7^ | 2.83×10^-6^ |
| GO:0012501 | programmed cell death | 1.78×10^-7^ | 2.88×10^-6^ |
| GO:1901653 | cellular response to peptide | 1.82×10^-7^ | 2.92×10^-6^ |
| GO:0015812 | gamma-aminobutyric acid transport | 1.83×10^-7^ | 2.92×10^-6^ |
| GO:0051953 | negative regulation of amine transport | 1.83×10^-7^ | 2.92×10^-6^ |
| GO:0040007 | growth | 1.85×10^-7^ | 2.96×10^-6^ |
| GO:0045927 | positive regulation of growth | 1.89×10^-7^ | 3.01×10^-6^ |
| GO:0009166 | nucleotide catabolic process | 2.04×10^-7^ | 3.23×10^-6^ |
| GO:0042176 | regulation of protein catabolic process | 2.06×10^-7^ | 3.24×10^-6^ |
| GO:0007417 | central nervous system development | 2.06×10^-7^ | 3.24×10^-6^ |
| GO:0019752 | carboxylic acid metabolic process | 2.11×10^-7^ | 3.30×10^-6^ |
| GO:0042327 | positive regulation of phosphorylation | 2.11×10^-7^ | 3.30×10^-6^ |
| GO:1901137 | carbohydrate derivative biosynthetic process | 2.30×10^-7^ | 3.58×10^-6^ |
| GO:0048659 | smooth muscle cell proliferation | 2.40×10^-7^ | 3.74×10^-6^ |
| GO:0045664 | regulation of neuron differentiation | 2.44×10^-7^ | 3.79×10^-6^ |
| GO:1901615 | organic hydroxy compound metabolic process | 2.45×10^-7^ | 3.79×10^-6^ |
| GO:0014048 | regulation of glutamate secretion | 2.53×10^-7^ | 3.88×10^-6^ |
| GO:0005996 | monosaccharide metabolic process | 2.54×10^-7^ | 3.90×10^-6^ |
| GO:0033762 | response to glucagon | 2.64×10^-7^ | 4.04×10^-6^ |
| GO:0060079 | excitatory postsynaptic potential | 2.66×10^-7^ | 4.05×10^-6^ |
| GO:0008202 | steroid metabolic process | 2.66×10^-7^ | 4.05×10^-6^ |
| GO:2000322 | regulation of glucocorticoid receptor signaling pathway | 3.07×10^-7^ | 4.66×10^-6^ |
| GO:0031331 | positive regulation of cellular catabolic process | 3.11×10^-7^ | 4.69×10^-6^ |
| GO:0006195 | purine nucleotide catabolic process | 3.11×10^-7^ | 4.69×10^-6^ |
| GO:0050769 | positive regulation of neurogenesis | 3.45×10^-7^ | 5.19×10^-6^ |
| GO:0071383 | cellular response to steroid hormone stimulus | 4.22×10^-7^ | 6.31×10^-6^ |
| GO:0006869 | lipid transport | 4.22×10^-7^ | 6.31×10^-6^ |
| GO:0045981 | positive regulation of nucleotide metabolic process | 4.54×10^-7^ | 6.75×10^-6^ |
| GO:1900544 | positive regulation of purine nucleotide metabolic process | 4.54×10^-7^ | 6.75×10^-6^ |
| GO:0031958 | corticosteroid receptor signaling pathway | 4.60×10^-7^ | 6.78×10^-6^ |
| GO:1900271 | regulation of long-term synaptic potentiation | 4.60×10^-7^ | 6.78×10^-6^ |
| GO:0035637 | multicellular organismal signaling | 4.60×10^-7^ | 6.78×10^-6^ |
| GO:0051934 | catecholamine uptake involved in synaptic transmission | 4.94×10^-7^ | 7.21×10^-6^ |
| GO:0051583 | dopamine uptake involved in synaptic transmission | 4.94×10^-7^ | 7.21×10^-6^ |
| GO:0042428 | serotonin metabolic process | 4.94×10^-7^ | 7.21×10^-6^ |
| GO:0000060 | protein import into nucleus, translocation | 4.96×10^-7^ | 7.21×10^-6^ |
| GO:0043113 | receptor clustering | 4.96×10^-7^ | 7.21×10^-6^ |
| GO:0006006 | glucose metabolic process | 5.08×10^-7^ | 7.36×10^-6^ |
| GO:0055080 | cation homeostasis | 5.40×10^-7^ | 7.79×10^-6^ |
| GO:1901565 | organonitrogen compound catabolic process | 5.40×10^-7^ | 7.79×10^-6^ |
| GO:1903510 | mucopolysaccharide metabolic process | 5.53×10^-7^ | 7.94×10^-6^ |
| GO:1901292 | nucleoside phosphate catabolic process | 5.53×10^-7^ | 7.94×10^-6^ |
| GO:0035773 | insulin secretion involved in cellular response to glucose stimulus | 5.63×10^-7^ | 8.06×10^-6^ |
| GO:0098657 | import into cell | 5.76×10^-7^ | 8.22×10^-6^ |
| GO:0032868 | response to insulin | 6.61×10^-7^ | 9.41×10^-6^ |
| GO:0001101 | response to acid chemical | 7.19×10^-7^ | 1.02×10^-5^ |
| GO:0001932 | regulation of protein phosphorylation | 7.41×10^-7^ | 1.05×10^-5^ |
| GO:0090493 | catecholamine uptake | 7.60×10^-7^ | 1.07×10^-5^ |
| GO:0090494 | dopamine uptake | 7.60×10^-7^ | 1.07×10^-5^ |
| GO:0007212 | dopamine receptor signaling pathway | 7.70×10^-7^ | 1.08×10^-5^ |
| GO:2000377 | regulation of reactive oxygen species metabolic process | 8.20×10^-7^ | 1.15×10^-5^ |
| GO:0071322 | cellular response to carbohydrate stimulus | 8.22×10^-7^ | 1.15×10^-5^ |
| GO:0050709 | negative regulation of protein secretion | 8.40×10^-7^ | 1.17×10^-5^ |
| GO:0098771 | inorganic ion homeostasis | 8.45×10^-7^ | 1.17×10^-5^ |
| GO:0045666 | positive regulation of neuron differentiation | 8.45×10^-7^ | 1.17×10^-5^ |

^a^ FDR:false discovery rate, which were calculated by the method of Benjamini & Hochberg (BH).
